# Quantitative Evaluation of OpenCV Visual Tracking Algorithms for Drawn Markers Under Dynamic Facial Deformation

**DOI:** 10.3390/s26206653

**Published:** 2026-10-21

**Authors:** Konrad Tkaczyk, Robert Sitnik

**Affiliations:** Institute of Micromechanics and Photonics, Faculty of Mechatronics, Warsaw University of Technology, ul. Św. Andrzeja Boboli 8, 02-525 Warsaw, Poland; konrad.tkaczyk7@gmail.com

**Keywords:** visual tracking, passive markers, tracking evaluation, non-rigid surface deformation, OpenCV, tracking robustness, computer vision, CSRT, DaSiamRPN, ViT

## Abstract

This study evaluates the eleven visual tracking algorithms available in OpenCV for tracking hand-drawn markers on deforming human faces. Using 41,641 multi-camera frames of seven subjects performing exaggerated phonetic and emotional movements, we assess each tracker’s deviation from an automated Blob Detection reference (four-marker sequences), its long-term marker identity retention (forty-marker sequences), and its throughput. Of the tested classical trackers, the Channel and Spatial Reliability Tracker (CSRT) performed best, retaining 96.2% of markers with a mean deviation of 1.94±1.29 pixels (approximately 0.46±0.31 mm) at 56.9 frames per second (FPS). The Kernelized Correlation Filter (KCF) matched this deviation at 445.4 FPS but failed under transient self-occlusion, retaining only 64.1%. Among the neural trackers, the Vision Transformer-based tracker (ViT) recorded the smallest deviation (1.06±1.03 pixels, approximately 0.25±0.25 mm) at 164.1 FPS, and the Distractor-Aware Siamese Region Proposal Network (DaSiamRPN) had the highest retention (92.4%, 1.35±1.26 pixels) at 11.6 FPS. Differences among the leading trackers were not significant at the subject level. The Minimum Output Sum of Squared Error (MOSSE) tracker reached about 12,500 FPS, but retained only 49.6%. Per-frame deviation and long-term retention are decoupled, meaning that both must inform tracker selection; scenario-based recommendations are provided.

## 1. Introduction

The precise acquisition and digital reconstruction of highly non-rigid dynamic surface deformations, including those exhibited by the human face, represent a fundamental challenge in optoelectronic metrology and biomechanical sensing [1,2]. While high-resolution static 3D coordinate acquisition has matured rapidly, capturing continuous high-fidelity spatial displacements in real time remains a key bottleneck due to the highly dynamic and non-rigid nature of facial tissue. In order to record these complex spatial coordinates, multi-camera optoelectronic tracking systems are widely deployed. While markerless computer vision approaches continue to advance [1,3], marker-based tracking frameworks [4] and hybrid multimodal configurations [5] remain the benchmark for high-precision applications [2,6], finding extensive use in gold-standard motion capture environments [7]. Recent research has focused heavily on evaluating industrial optoelectronic sensory pipelines [8] and their downstream integration into kinematic solvers [2,9]. However, traditional physical markers such as bulky retro-reflective spheres impose significant constraints. The spatial resolution of the coordinate grid is inherently limited, as these physical markers cannot be applied in high densities without altering the natural mechanical properties of the underlying skin or causing physical occlusions.

An alternative is drawing flat markers directly onto the skin of the subject. Painted skin patterns have been employed to render facial skin trackable in dense multi-view capture [10]. Sparse drawn dots constitute their marker-based counterpart, and are the passive epidermal markers evaluated in this work. Manual marking requires considerably less preparation time than adhesion protocols. Because drawn markers conform to the epidermal layer, they can translate skin deformation into an optical signal without adding mass or stiffness to the skin or obscuring the fine geometric detail that multi-scale capture pipelines seek to preserve [2]. In addition, their layout can follow the same density arguments that apply to physical markers [4]. Despite these advantages, to the best of our knowledge no systematic evaluation of tracking algorithms for hand-drawn markers within multi-camera acquisition frameworks has been reported; the closest prior work evaluated OpenCV trackers on rigid or sparsely occluded targets [11,12].

Within such a system, the tracking algorithm directly determines the spatial resolution, noise characteristics, and tracking stability of the entire measurement pipeline. Although numerous template-matching, filter-based, and deep-learning-based object tracking frameworks have been proposed [13,14,15,16] and open-source packages such as the OpenCV library [17] provide accessible implementations, a significant knowledge gap persists regarding their comparative performance when tracking dense and near-identical drawn epidermal markers on dynamic deforming human faces.

To address this gap, in this work we present a comprehensive evaluation of eleven computer vision tracking algorithms implemented within the open-source OpenCV framework [17]. We characterize the performance of these tracking algorithms along three operational dimensions: (1) deviation of the tracked marker centre from a reference trajectory under non-rigid deformation; (2) the temporal robustness of marker identity maintenance (indexing) across complex kinematic sequences; and (3) computational execution latency. Unlike prior OpenCV tracker comparisons that examined a subset of classical algorithms on rigid-body or sparse-target sequences [11,12], in this work we evaluate the complete set of eleven available OpenCV trackers, including the most recent additions (TrackerNano (2022) [18,19] and the ViT tracker (2023) [20], which is based on the Vision Transformer architecture [21]), under dense, non-rigid, and multi-target conditions with achromatic targets. These conditions expose failure modes such as identity swapping, occlusion recovery failure, and bounding box expansion that sparse or rigid-body benchmarks fail to capture, thereby demonstrating that per-frame deviation does not predict long-term tracking stability. Ultimately, this work aims to identify the best-performing configuration among the eleven evaluated OpenCV trackers, thereby establishing the practical boundaries of the trade-off between per-frame deviation, tracking stability, and computational throughput in optoelectronic facial displacement sensing.

This task differs from conventional single-object tracking in four respects that shape the evaluation protocol. First, up to forty near-identical targets are present in a single face; since these are achromatic, appearance alone cannot disambiguate them. Second, the targets deform non-rigidly, with no global rigid transformation between frames. Third, skin folds and lip contact can cause transient self-occlusion. Fourth, when a marker’s identity is silently exchanged with that of a neighbouring marker, it continues to report a plausible low-deviation trajectory while corrupting the downstream reconstruction; for this reason, identity retention is treated as a primary metric alongside per-frame deviation.

This work is a benchmark and evaluation study of continuous coordinate tracking for dense epidermal markers under non-rigid facial strain. Its contributions are fivefold: (1) a dataset of hand-drawn epidermal markers across multi-view dynamic sequences, with coordinate data released together the evaluation code and an open-source benchmark application by which the eleven trackers can be evaluated on other videos; (2) a documented evaluation protocol that pairs deviation conditional on active tracking with failure-timing measures, which shows that per-frame deviation and long-term retention are decoupled; (3) a per-algorithm taxonomy of failure modes (identity swapping, bounding box expansion, occlusion loss) across the eleven OpenCV trackers; (4) a scenario-based selection table derived from the above taxonomy; and (5) the design and evaluation of a cooperative hybrid pipeline (ViT+CSRT) that removes most of the identity swapping of the primary tracker at the cost of running both trackers.

## 2. Related Works

A key aspect of dynamic non-rigid surface reconstruction is the design and optimization of the coordinate acquisition pipeline, which is fundamentally governed by the physical markers, their spatial topology, and the digital tracking algorithms used to compute the final trajectories. This section reviews the state of the art in optical coordinate sensing, spatial sampling layouts, and the trackers that determine the quality of the reconstructed kinematic data.

### 2.1. Optoelectronic Coordinate Acquisition and Sensing Paradigms

The acquisition of dynamic human facial deformations is classically achieved by combining high-resolution static 3D geometric scans with real-time spatial displacement tracking [22]. Industrial pipelines (e.g., Infinite Realities [23], 3DMD [24]) utilize multi-camera arrays to capture dynamic geometry, which is subsequently processed via topological retopology [25] or non-rigid mesh optimization [26] to estimate continuous motion. Active optical sensing methods, including structured light projection [27], laser triangulation [28], and time-of-flight (ToF) [29], provide dense geometric coordinates, whereas photogrammetric methods [30] and shape-from-silhouette reconstructions [31] exploit passive visual cues. With the rapid advancement of digital image sensors, automated Structure from Motion (SfM) pipelines [32] have become a dominant paradigm. SfM systems offer high-fidelity textural registration and lower calibration complexity compared to alternative spatial sensing modalities such as magnetic or inertial measurement units (IMUs) [33], which are prone to electromagnetic interference and sensor drift. Consequently, multi-camera optoelectronic vision systems remain the gold standard for high-accuracy facial displacement sensing.

Within optical coordinate sensing, systems are categorized as anthropometric- based [34], neural network-based [35], or marker-based [36]. Despite the continuous development of markerless tracking algorithms, they still cannot match the spatial resolution, accuracy, and reliability of marker-based solutions under highly nonlinear or extreme deformations [35,37]. Leading commercial optoelectronic tracking platforms such as Vicon [38], OptiTrack [39], and Qualisys [40] offer high-frequency 3D coordinate estimation; however, comparative studies indicate that their performance (ranging from sub-millimetre to millimetre-level errors) is heavily dependent on the specific calibration volume, lighting, and experimental evaluation protocol [8,41,42]. Although these industrial platforms provide sufficient positioning accuracy for standard biomechanical and animation pipelines, their use is constrained by high costs, complex hardware synchronization demands with active 3D scanning systems, and the physical limits of retro-reflective marker density. Heavy retro-reflective spheres alter the natural viscoelastic properties of the skin and cause physical occlusions which prevent them from being deployed in dense configurations. Passive epidermal markers drawn directly on the skin address these limitations by conforming perfectly to the epidermal boundary, preserving micro-geometric features such as wrinkles and pores, and avoiding interference with active range-imaging sensors.

### 2.2. Spatial Sampling Topology and Marker Layouts

The spatial layout of markers on the non-rigid facial surface is a critical design parameter that defines the spatial sampling rate of the continuous deformation field. Empirical or heuristic layouts, while common in practical applications, often fail to capture complex localized strain fields. To optimize this process, several data-driven approaches have been proposed to determine the mathematically optimal layout and minimum number of tracking nodes; for instance, Reverdy et al. [43] developed an automated marker selection method based on unsupervised clustering of mesh vertex trajectories. While this approach outperformed standard empirical layouts such as MPEG4 (53 markers) [44], Face Robot (39 markers) [45], SignCom (41 markers) [46], and Sign3D (60 markers) [47] in terms of root mean square error (RMSE), it requires a prior database of dense 3D scans for each subject and exhibits high computational complexity.

**Figure 1:**
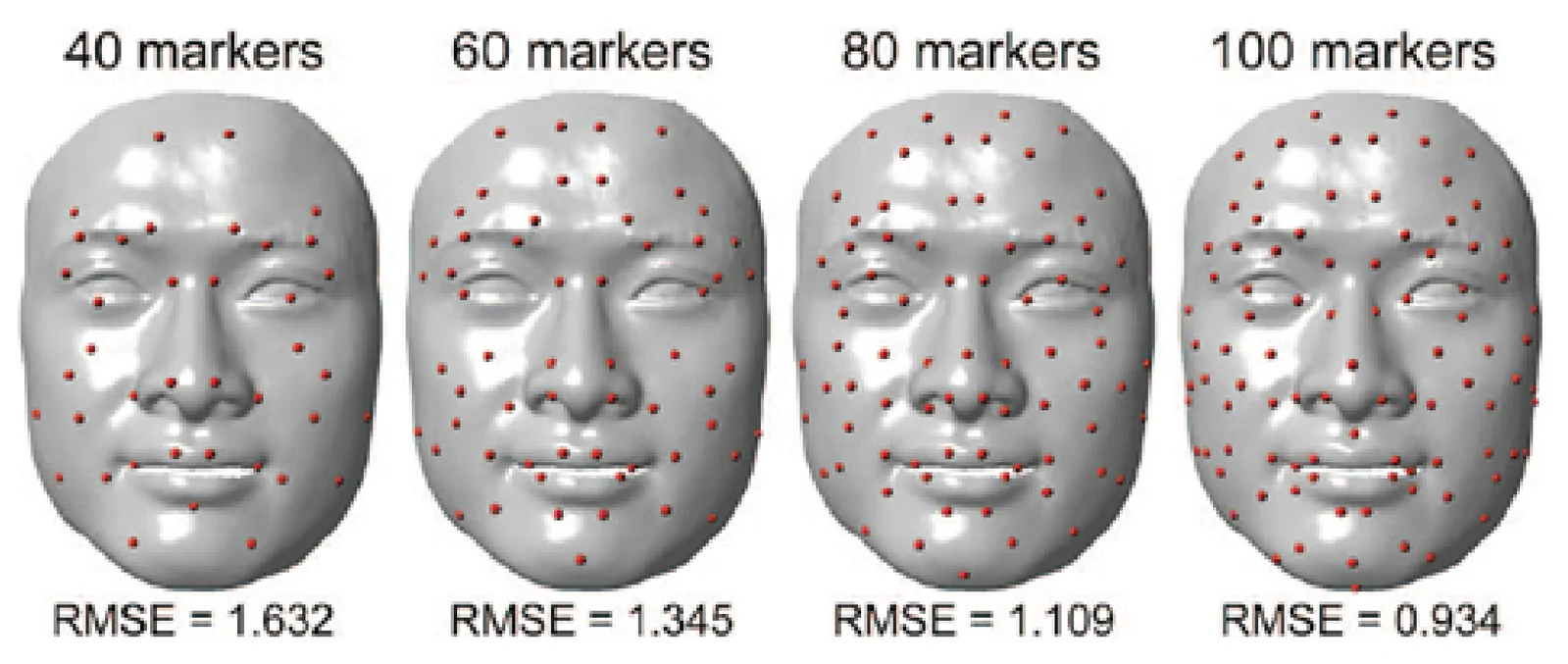
Different facial marker layouts and their millimetre RMSE values [4].

A more generalized optimization framework was established by Le et al. [4], who optimized the spatial distribution of tracking nodes by minimizing the RMSE of a marker-driven linear deformation model. This study demonstrated that the reconstruction error decreases monotonically as the number of markers increases, as illustrated in Figure 1. Crucially, it provides a mathematical foundation for choosing the optimal trade-off between the physical marker density and the target reconstruction fidelity, allowing for custom layouts tailored to the subject’s unique anatomical and muscle activation patterns.

### 2.3. Tracking Algorithms

In an optoelectronic tracking system, the tracking algorithm transforms temporal changes in pixel intensity into continuous coordinate streams. Over the past decades, visual tracking has evolved across multiple algorithmic paradigms, including mean shift tracking [48], recursive filter-based state estimation [49], optical flow estimation [50], template matching [14], and deep learning-based regression [51]. The mean shift algorithm [52] tracks non-rigid objects by iteratively shifting a kernel toward the local peak of a probability distribution, but struggles with rapid abrupt motions. Filter-based tracking, typically utilizing the Kalman filter [53], models the target’s kinematic state to filter out background noise; it achieves high execution speeds, but relies heavily on accurate motion models. Optical flow methods [54] compute pixel-level velocity fields between consecutive frames to estimate coordinate displacement; however, they are highly sensitive to illumination variations. Template matching [55], which is widely used in augmented reality [56], offers high robustness by matching geometric features across frames, although it is prone to drift under non-rigid deformation. Recently, deep learning-based trackers have achieved superior performance by learning robust target representations from large-scale video datasets [57] or by dynamically modeling the spatiotemporal relationship between target appearance and motion [58].

To evaluate these tracking mechanisms within an accessible open-source framework, this study focuses on the eleven tracking algorithms implemented in the OpenCV library [17]. The oldest method, Boosting [59], uses an online AdaBoost framework to train a discriminative classifier frame-by-frame, creating a confidence map to update the target’s bounding box. The Multiple Instance Learning (MIL) tracker [60] improves upon this by grouping training samples into positive and negative bags, which mitigates the drift caused by slight misalignment in positive samples. Comparative benchmarks of these early classifiers show similar performance profiles under stable conditions [11]. The Tracking–Learning–Detection (TLD) framework [61] decouples the process into three parallel modules: a median flow tracker [62] to estimate short-term frame-to-frame motion, a detector to localize the target on each frame independently, and a learning module that continuously updates the detector’s classifier. To achieve higher computational throughput, correlation filter-based trackers have been widely adopted. The Kernelized Correlation Filter (KCF) [63] improves upon the MIL framework by exploiting the circulant matrix structure of translated image patches, computing ridge regression in the dual space via the Fast Fourier Transform (FFT) [64]. Similarly, the Minimum Output Sum of Squared Error (MOSSE) tracker [65] uses FFT-based correlation filters to minimize the squared error between actual and desired correlation outputs, resulting in exceptional frame rates on low-power CPUs. However, classical correlation filters struggle with non-rigid target deformations and boundary drift. The Channel and Spatial Reliability Tracker (CSRT) [66] addresses this by introducing spatial and channel reliability maps, which dynamically constrain the filter’s spatial support to the most stable regions of the target, solved efficiently as a graph-labeling problem in each frame.

OpenCV also integrates deep learning-based trackers. The GOTURN tracker [58] employs a deep convolutional neural network (CNN) trained offline to regress the target’s bounding box coordinates directly from one frame to the next. To enhance tracking under complex distractions and occlusions, the Distractor-Aware Siamese Region Proposal Network (DaSiamRPN) [67], one of several Siamese-based tracking architectures [68], incorporates semantic background information during offline training, dynamically updating its template model to suppress similar-looking background distractors. The TrackerNano (2022) algorithm [18], a lightweight Siamese tracker derived from LightTrack [19] and SiamBAN [69], combines a compact feature extraction backbone with a lightweight localization head (Neckhead). The most recent addition is the ViT tracker, which is based on the Vision Transformer architecture [21]; the specific model evaluated here is the VitTrack model distributed through the OpenCV Zoo [20], contributed to OpenCV in 2023, for which no peer-reviewed description of the model, its training data, or its weights exists, so the results reported here characterise the distributed model. By replacing traditional convolutional layers with multi-head self-attention mechanisms, ViT models the global relationship between target patches, enabling superior bounding box adaptation and spatial accuracy.

These tracking frameworks have been evaluated on general computer vision benchmarks [11] by comparing classical OpenCV trackers and cataloguing the principal failure conditions they encounter. More recently, a domain-specific study [12] compared seven OpenCV trackers on ultrasound video sequences for liver tumor tracking, identifying CSRT and KCF as the leading algorithms in terms of both accuracy and real-time performance. While their evaluation methodology aligns with our approach and demonstrates the value of domain-specific OpenCV benchmarking, their target represents a single sparsely-occluded and relatively localized structure, and the most recent OpenCV trackers (TrackerNano, ViT) were absent from their evaluation.

To date, no study has characterized the spatial tracking deviation relative to a reference trajectory, temporal indexing stability, and computational latency of these algorithms when tracking dense and near-identical hand-drawn markers on dynamically deforming human faces. The present study addresses this gap through a domain-specific benchmark of all eleven currently available OpenCV trackers.

The set of trackers available in OpenCV lags behind the current state of the art in visual object tracking, as recent surveys of discriminative filter, Siamese, and transformer-based trackers have documented in detail [70,71,72]. Nonetheless, we take it as the object of this study because it represents what practitioners can deploy from a stable and permissively licensed CPU-capable library without training infrastructure. Section 6.2 places the present dataset against the benchmarks on which the newer trackers are evaluated.

Table 1 provides a structured overview of all evaluated OpenCV tracking algorithms, grouped by their underlying methodology.

### 2.4. Summary

The design of an optoelectronic facial displacement sensing system involves a multi-dimensional optimization problem across three coupled stages: selecting the physical marker transduction medium, determining the spatial sampling node topology, and deploying the tracking algorithm. While commercial systems are widely used, their high cost, physical marker size limits, and synchronization constraints make low-cost dense passive epidermal markers an attractive alternative. However, selecting and configuring the best-suited tracking algorithm to process these passive coordinate streams remains an open research question, which this work systematically addresses.

## 3. Materials

This section details the experimental and hardware framework utilized in this study, including the biomechanical cohort, the multi-camera optoelectronic spatial sensing array, the dynamic deformation acquisition protocol, and the physical configuration of the passive epidermal markers. These components were integrated in order to rigorously benchmark the performance of the eleven visual tracking algorithms using a high-fidelity 4D spatial coordinate acquisition system.

### 3.1. Human Subjects

**Figure 2:**
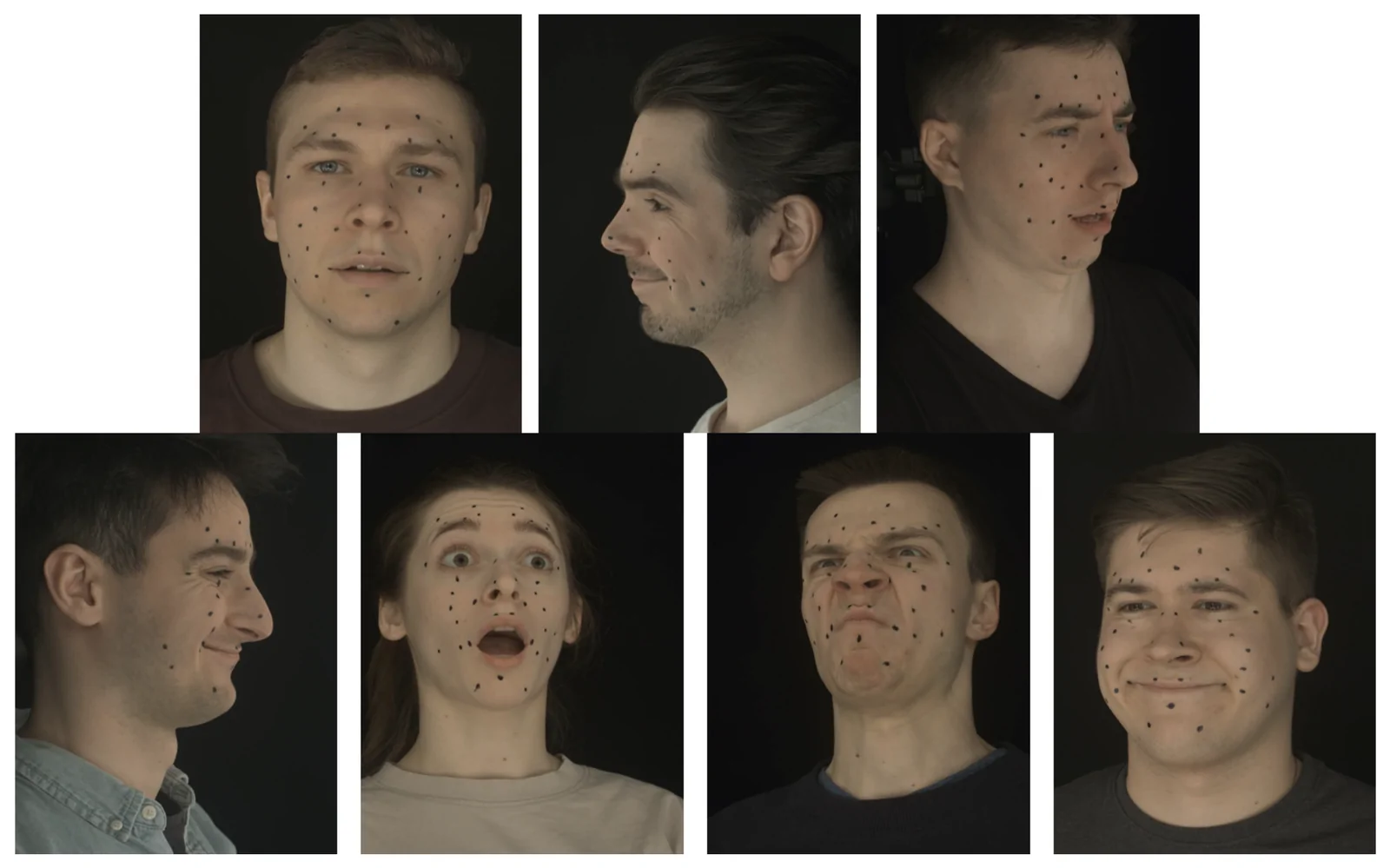
Depictions of all participants with various poses and camera angles.

To evaluate the tracking algorithms, a cohort of seven healthy human subjects (six males and one female, aged 23 to 28 years) of Central European descent was selected. The research was conducted in strict accordance with the Declaration of Helsinki, and informed consent was formally obtained from all participants prior to the measurements. The experimental subjects and their diverse geometric poses are documented in Figure 2.

### 3.2. Multi-Sensor 4D Spatial Coordinate Acquisition Array

**Figure 3:**
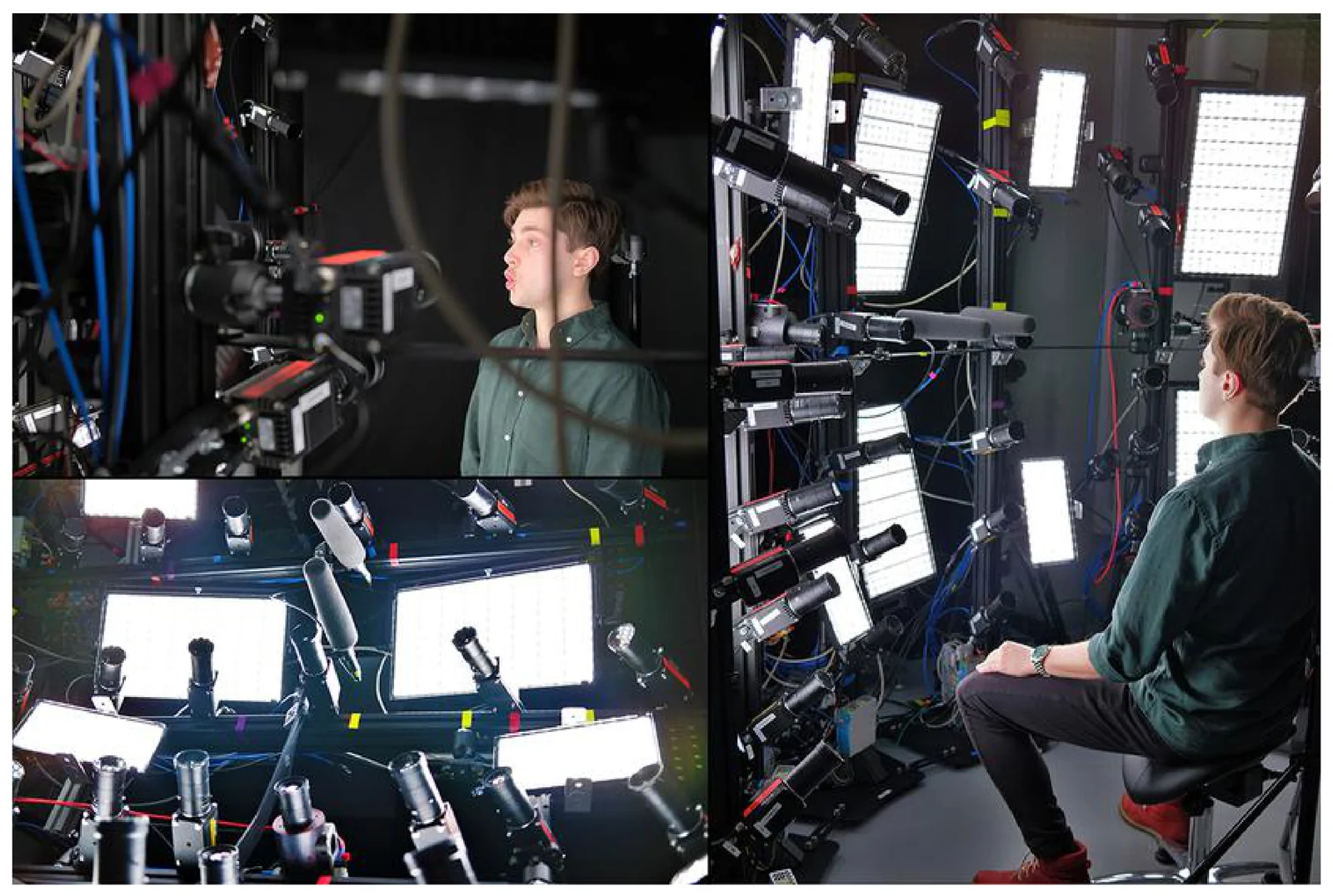
The Mnemosis system for 4D facial scanning. Image courtesy of Mnemosis [73].

The optoelectronic sensing framework employed for dynamic coordinate acquisition is a custom high-density 4D scanning system developed by Mnemosis (Warsaw, Poland) [73]. The sensor topology comprises 32 industrial RGB Oryx cameras (Teledyne FLIR LLC, Wilsonville, OR, USA) equipped with high-resolution CMOS sensors featuring a native resolution of 4096×2160 pixels. For computational efficiency during the software-level tracking evaluation, the raw video streams were spatially downsampled to a processing resolution of 1920×1080 pixels. The active lighting setup consists of a synchronized array of high-CRI LED panels (Akurat Lighting, Pruszków, Poland) designed to provide uniform diffuse illumination, minimise dynamic self-shadowing, and maximize the signal-to-noise ratio (SNR) around the passive marker regions. Hardware-level wired synchronization ensures zero inter-camera shutter latency. The multi-camera array was geometrically calibrated using a planar calibration target, achieving a global mean reprojection error of 0.2 pixels or less. The physical architecture of this multi-sensor acquisition system is illustrated in Figure 3.

### 3.3. Experimental Kinematic Protocol and Acquired Dataset

To challenge tracking algorithms under realistic and highly nonlinear surface strain fields, each subject performed two distinct dynamic kinematic sequences. The first sequence, designated “Emotions”, comprised four consecutive highly exaggerated facial expressions designed to induce maximum non-rigid surface stretching and compression. The second sequence, designated “Visemes”, consisted of ten phonetically distinct vocalizations selected from the standardized Viseme Reference [74], as detailed in Table 2.

These sequences were chosen to span a wide range of natural facial motion. Subjects were instructed to exaggerate all movements so as to induce rapid, transient displacements, which are the most challenging conditions for a tracker. Captured dynamic deformation sequences provide a vital foundation for modern spatial coordinate solvers, a paradigm that builds on early mathematical formulations of morphing visual speech cues [75].

### 3.4. Passive Marker Configuration and Morphological Calibration

As established in Section 2.2, increasing the spatial density of tracking nodes reduces surface reconstruction error, although it concurrently increases the risk of multi-target index confusion and spatial cross-talk. To address these competing factors, the experimental design decouples the evaluation into two specific testing pipelines:

1. Centring Deviation Pipeline (Low-Density): To isolate the centring behaviour of the tracking algorithms, a low-density subset of four hand-drawn markers was applied to the subject’s face. This configuration minimizes the risk of temporal indexing failures, ensuring that the computed Euclidean distances purely reflect the deviation of the tracking algorithm from the reference.

2. Indexing Robustness Pipeline (High-Density): To evaluate temporal state maintenance under dense and complex spatial topologies, a high-density grid of forty hand-drawn markers was applied. This spatial layout was adopted from the mathematically optimized data-driven topology presented in Figure 1, representing a balance between spatial sampling density and operational feasibility.

The two pipelines differ by design. The four-marker layout keeps the targets widely separated, so that the measured deviation reflects centring behaviour alone without mis-association, whereas the forty-marker layout places the targets in close proximity so that identity retention is stressed. Measuring both quantities on a single layout would confound them; the consequences for their joint interpretation are discussed in Section 5.4.

**Figure 4:**
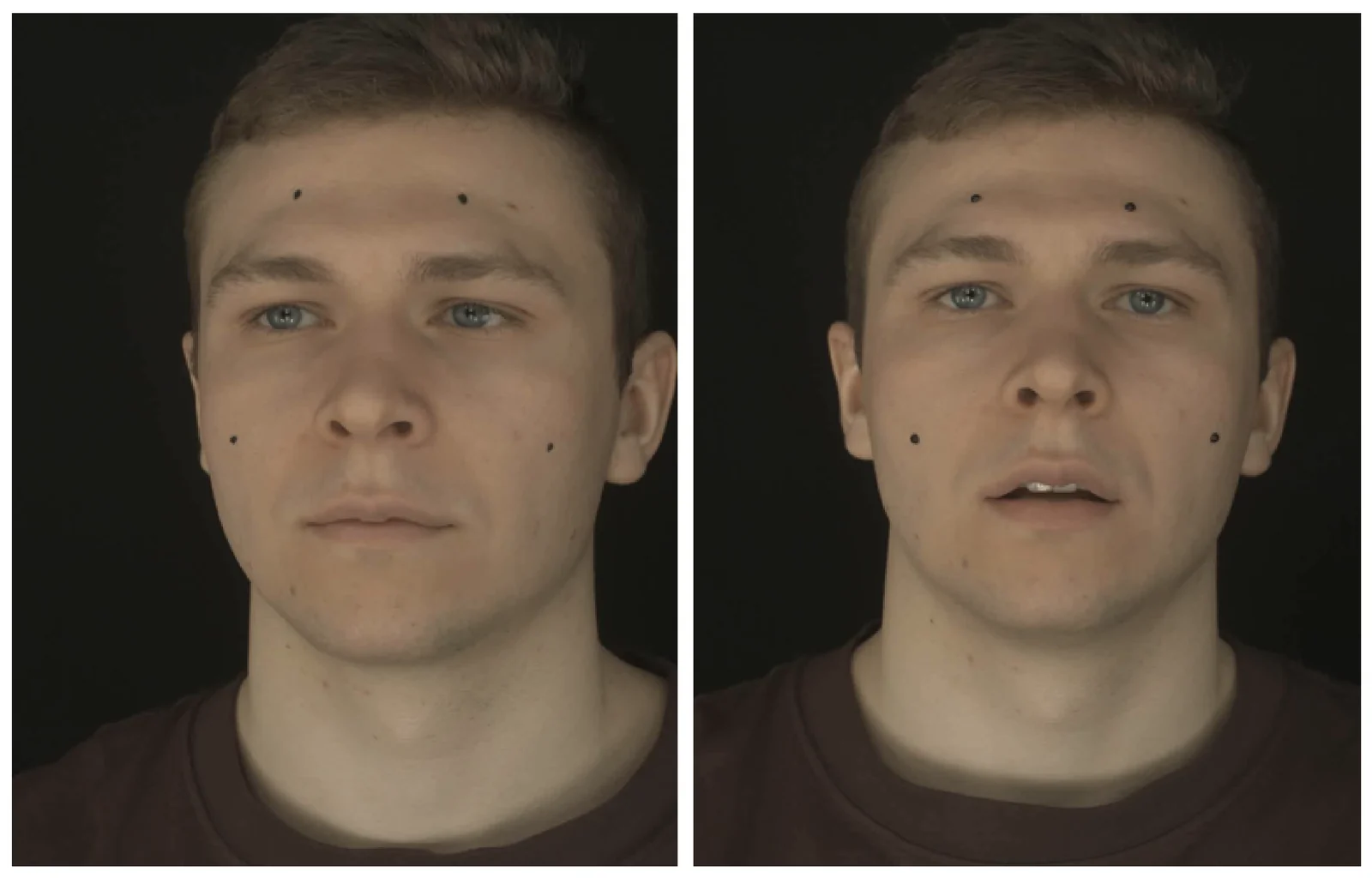
Arrangement of four evaluation markers. On the **left**: drawn; on the **right**: semi-spherical glued.

Hand-drawn passive epidermal markers conform perfectly to the skin’s surface without altering its local viscoelastic properties. However, manual application introduces slight morphological anisotropy (irregular boundaries) and local shape variations during muscle contraction. To quantify this morphological effect, a reference dataset was captured using rigid semi-spherical black markers 3 mm in diameter, which were physically adhered to the epidermal layer to provide a geometrically invariant control reference (Figure 4).

In total, each of the seven participants executed the two dynamic kinematic sequences (“Emotions” and “Visemes”) across the two physical marker configurations (four drawn markers, forty drawn markers). Additionally, for reference purposes, one person performed two recordings with four glued semi-spherical reference markers. This experimental grid yielded thirty dynamic multi-camera recordings (fourteen with four drawn markers, fourteen with forty drawn markers, and two with glued reference markers) captured simultaneously by the 32-camera array, providing a robust empirical foundation for the subsequent benchmarking analysis.

## 4. Methods

**Figure 5:**
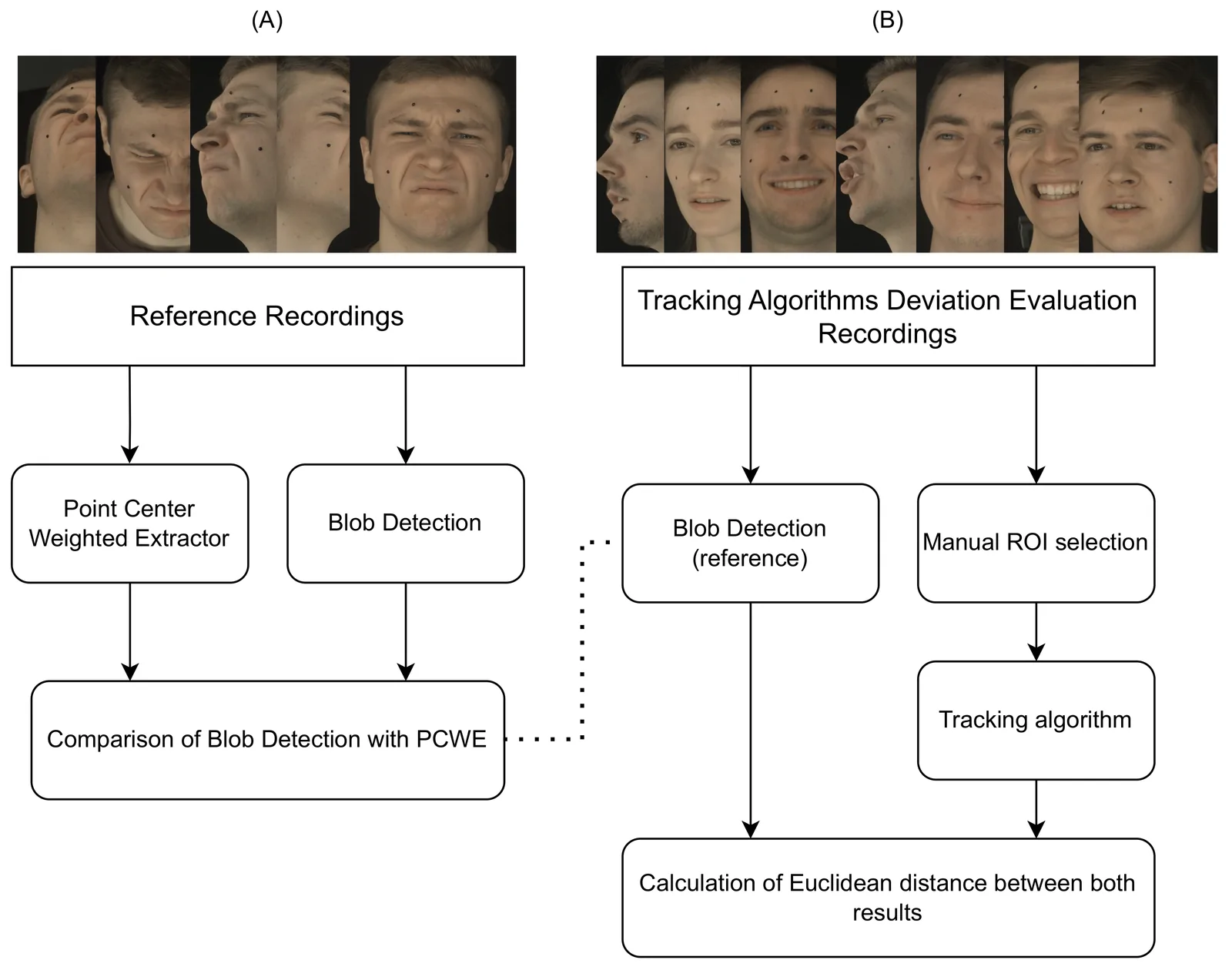
Evaluation protocol schemes: (**A**) protocol path for comparing Blob Detection with PCWE and (**B**) processing steps for evaluating the deviation of the tracking algorithms from the reference.

Under the controlled boundary conditions of the optoelectronic coordinate acquisition setup, several classical tracking perturbations (e.g., severe illumination transitions, scale variations, and camera egomotion) are minimized [11]. Thus, the primary non-rigid sensing challenges were refined to three distinct phenomena: (1) transient self-occlusions, (2) morphological deformations under high local surface strain, and (3) localized background clutter arising from dynamic skin micro-features (e.g., wrinkles and transient facial furrows). To address these challenges, this study establishes a benchmarking framework focused on three core performance dimensions: the deviation of the tracked marker centre from the reference, the temporal robustness of marker identity indexing, and the computational execution latency. The evaluation protocols are illustrated schematically in Figure 5.

### 4.1. Quantitative Evaluation of Reference Coordinate Extraction

To establish a high-throughput automated reference coordinate pipeline and compile a robust benchmarking dataset comprising 41,641 frames, the multi-extremum OpenCV Blob Detection algorithm was utilized [17]. For each frame, the Blob Detection algorithm was executed with the parameter values listed in Appendix A to extract the (x,y) spatial coordinates of the tracking nodes. The resulting coordinates serve as the baseline trajectory against which all other trackers are compared. By calculating the Euclidean distance between the reference centre and the centre coordinates estimated by each visual tracker, the deviation from the reference is quantified.

Since the baseline Blob Detection algorithm may introduce sub-pixel coordinate deviations, its centroids were compared with a second sub-pixel extraction algorithm, designated as the Point Center Weighted Extractor (PCWE).

#### Point Center Weighted Extractor (PCWE)

**Figure 6:**
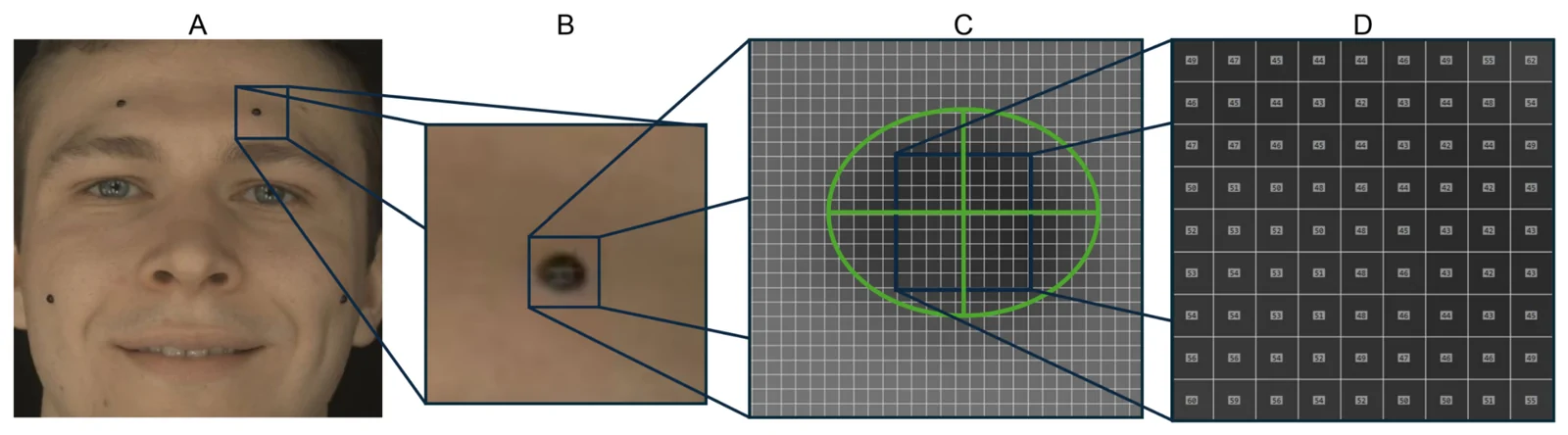
Schematic overview of the Point Center Weighted Extractor, shown as a left-to-right zoom-in sequence: (**A**) input frame containing glued facial markers, (**B**) local region of interest cropped around one marker after coarse blob detection and mask dilation, (**C**) extracted marker contour (green outline) together with the resulting sub-pixel centre, indicated by the intersection of the green crosshairs, and (**D**) individual pixels inside the marker patch with their intensity values. Lower values indicate darker areas, and hence higher weights. The weighted centroid of these pixels gives the refined sub-pixel centre shown in (**C**).

The PCWE algorithm serves as the control used to assess the agreement of the high-throughput Blob Detection reference pipeline. The multi-stage processing pipeline of the PCWE is schematically detailed in Figure 6 and proceeds through three sequential phases:

1. Coarse Localization and Spatial Masking: High-frequency sensor noise is mitigated by applying a Gaussian filter with an 11×11 pixel kernel. Coarse centroid coordinates are then extracted via baseline blob detection. Concurrently, a local binary mask is generated around each coarse center by executing inverse Otsu thresholding combined with a flood-fill operation seeded at the detected center. This mask is subsequently dilated three times with a 5×5 pixel elliptical structuring element to constrain all subsequent low-level pixel operations strictly to the local marker neighborhood.
2. Differential Boundary Extraction: An adaptive edge detection operator is applied within the localized Region of Interest (ROI). For each pixel, the mean absolute intensity difference relative to its circular neighborhood kernel is calculated and compared against a dynamically computed threshold to isolate the high-contrast boundary edge pixels of the marker. The resulting binary edge map is masked using the dilated ROI from the first stage, retaining only the precise outer contours of the passive epidermal marker.
3. Intensity-Weighted Centroid Refinement: The external boundary contours are extracted from the masked edge map and matched with their corresponding coarse centers. For each matched contour *C*, the refined sub-pixel center coordinates $c=(c_{x},c_{y})$ are computed using an intensity-weighted centroid formulation:

(1) $$c=\frac{\sum_{p\in C}w_{p}p}{\sum_{p\in C}w_{p}}$$

where $p=(x_{p},y_{p})$ is the spatial coordinate vector of a boundary contour pixel. The weighting function is defined as

(2) $$w_{p}=1-\frac{I_{p}-I_{\min}}{I_{\max}-I_{\min}},$$

where $I_{p}$ is the localized grayscale intensity of the pixel, while $I_{\min}$ and $I_{\max}$ are the minimum and maximum pixel intensities within the local marker patch surrounding contour *C*.

PCWE minimizes the effect of morphological boundary irregularities and skin reflectance variations by assigning higher weights to darker marker-like pixels, yielding sub-pixel global coordinates.

For the 32-camera Flir Oryx array, which records at a native resolution of 4096×2160 pixels (downsampled to 1920×1080 pixels for digital signal processing), the geometric calibration of the Mnemosis scanning volume yields an effective spatial image scale of approximately 0.24 mm/pixel at the target facial surface. This is a single nominal value for a non-planar surface imaged at varying depth; millimetre quantities derived from it are approximate image-scale conversions. The nominal factor is applied to translate pixel-domain Euclidean deviations (Equation (3)) into millimetres.

**Figure 7:**
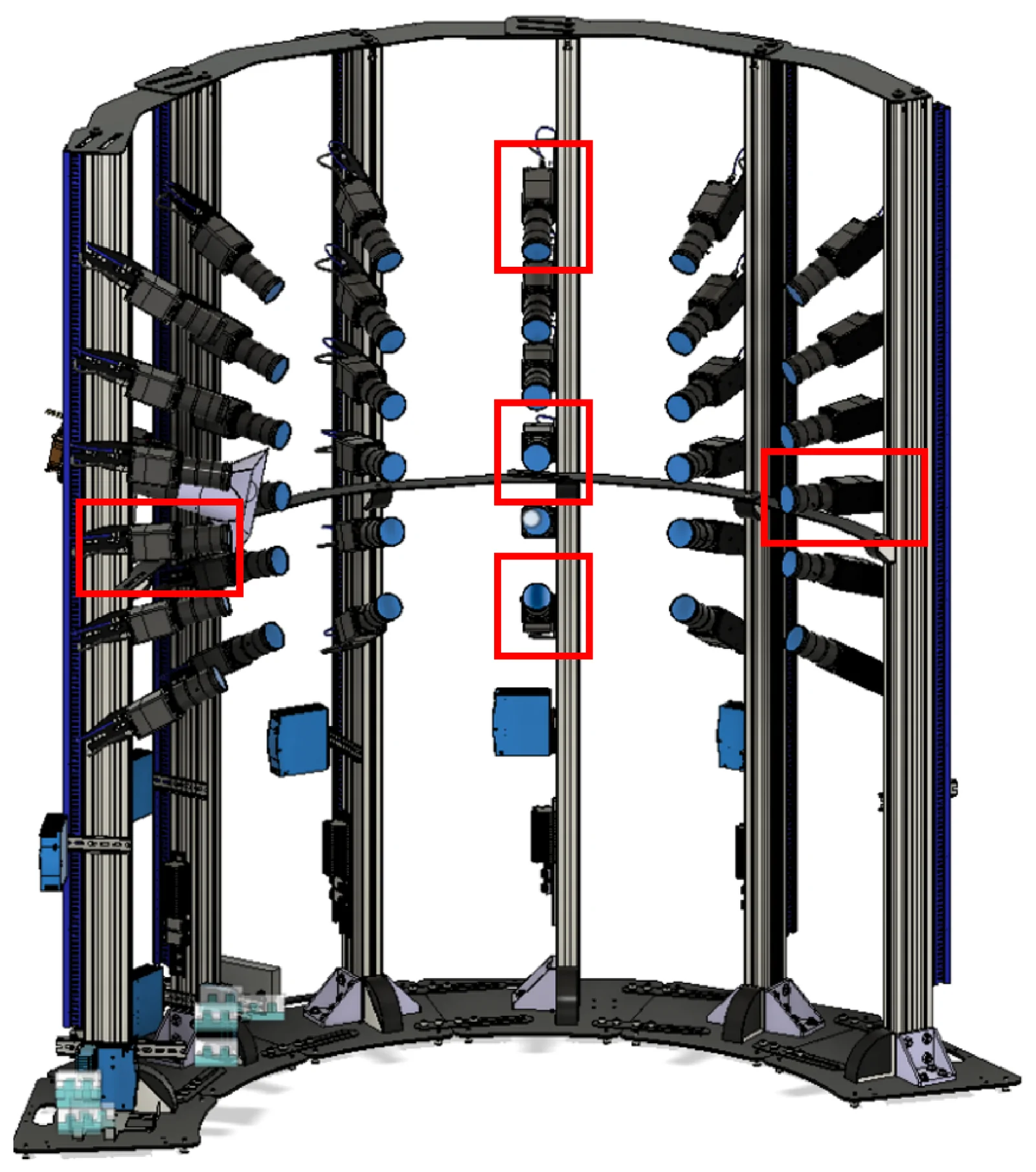
Schematic of the multi-camera optoelectronic system showing the camera viewpoints selected for the evaluation. The red rectangles mark the selected cameras.

To evaluate the agreement of the Blob Detection pipeline with the PCWE control, both extraction algorithms were executed on a continuous measurement sequence containing 3 mm physical semi-spherical markers. The benchmark was conducted across five synchronized camera viewpoints, capturing the calibration volume from extreme angles: left, right, top, bottom, and front, as illustrated in Figure 7.

The glued semi-spherical markers were chosen for this comparison because their rigid, circular, and high-contrast boundary provides a stable geometric target on which the centroid estimate of the detector can be assessed independently of marker deformation. The PCWE shares the coarse localisation stage of the Blob Detection pipeline and refines the centre within the local marker neighbourhood; in this way, the comparison quantifies the agreement of the two centroid estimates, i.e., how far the detector centroid departs from an intensity-weighted sub-pixel centre. The same comparison was additionally carried out on the hand-drawn markers during dynamic deformation (Section 5.1), so that the influence of deformation and of irregular marker boundaries on the centroid estimate is quantified directly. Because one reference trajectory is used for every tracker, the evaluation is a comparison between trackers under a common reference, and the reported quantities are accordingly described as deviations from the reference.

Because the PCWE shares the coarse localisation stage of the Blob Detection pipeline, the reference was also compared with manual annotation, which shares no processing step with either extractor. Two annotators independently marked the centre of the drawn marker in enlarged image patches, which were cut around the marker with a random offset and shown without any detector output. The sample comprised every marker of the four-marker sequences in all five viewpoints and ten randomly chosen markers of each forty-marker sequence, each in a frame near the start of the sequence and in the frame of its largest displacement, together with 100 marker-frames in which the Blob Detection and PCWE positions differed by more than two pixels or only one of them located the marker; 10% of the patches were shown twice to measure the repeatability of each annotator. The manual centre is the mean of the two annotators’ marks.

### 4.2. Deviation-from-Reference Evaluation Pipeline

To evaluate the deviation of the eleven visual tracking algorithms from the reference, the automated Blob Detection trajectory was used as the high-throughput coordinate reference. The evaluation was performed across five camera viewpoints at extreme spatial angles, capturing the dynamic sequences (“Visemes” and “Emotions”) of all seven subjects using the four-marker physical configuration, yielding a dataset of 70 high-definition video sequences.

Initially, each recording was processed using the reference Blob Detection algorithm to extract reference trajectories $\left(X_{\mathrm{BD}},Y_{\mathrm{BD}}\right)$.

The initial bounding box of each marker was defined once by a single operator in the first frame, with the same set of boxes used for every tracker so that all trackers were initialised from identical regions; the boxes measured approximately three times the marker diameter. The tracking algorithms were then executed to compute the estimated coordinate trajectories $\left(X_{\mathrm{TR}},Y_{\mathrm{TR}}\right)$. All algorithms were initialized with their default parameters. For the correlation filter trackers, previous work has shown that manual tuning of initialization parameters yields no significant gain while risking overfitting [12]. The neural trackers in OpenCV (GOTURN, DaSiamRPN, Nano and ViT) are fixed pretrained models in which the parameter structures expose model paths and the inference backend, but do not expose any tunable tracking hyper-parameters; thus, the only initialisation choice common to all eleven trackers is the initial region, the influence of which is examined in Section 5.3.

For each marker and each frame *i* in which the tracking state remained active, the instantaneous spatial deviation from the reference was computed as the Euclidean distance:

(3) $$d_{i}=\sqrt{{\left(X_{\mathrm{TR},i}-X_{\mathrm{BD},i}\right)}^{2}+{\left(Y_{\mathrm{TR},i}-Y_{\mathrm{BD},i}\right)}^{2}}.$$

This deviation was calculated for each frame, and the resulting distributions were compiled for each tracker.

Equation (3) was evaluated only on frames in which the tracker had not lost its marker under the failure conditions of Section 4.3 and where a reference position existed, so the resulting mean is conditional on active tracking. A mean computed over a small surviving subset of frames is not comparable with one computed over a whole sequence, so the fraction of frames on which each tracker remained active is reported with it in Section 5.2 and with the region size analysis of Section 5.3. The component deviations $d_{x}$ and $d_{y}$ are reported as mean absolute values.

Per-frame observations are autocorrelated and nested within markers, views, sequences, and subjects. The dispersion reported with each mean deviation is pooled over frames, and describes the frame-to-frame spread of a tracker. Uncertainty at the level of an independent experimental unit is reported separately in Section 5.2; the deviations of the frontal-view trajectories are aggregated hierarchically, from frames to trajectories and from trajectories to subjects, and a 95% percentile bootstrap confidence interval is computed over the seven subjects. The frontal view is used because it is the only viewpoint in which all four markers are visible in every sequence, meaning that every subject contributes the same markers. Differences between trackers are tested on these subject means: a Friedman test checks whether the trackers differ at all, and Kendall’s coefficient of concordance *W* measures how consistently the seven subjects rank them (0, no agreement; 1, identical rankings). Each pair of trackers within the leading tier and each pair adjacent in the order of mean deviation is compared by a paired percentile bootstrap interval of the per-subject differences and by an exact two-sided sign-flip permutation test, with the *p*-values Holm-corrected for the number of tested pairs.

### 4.3. Temporal State Maintenance and Multi-Target Identity Verification

To evaluate the temporal robustness of the tracking algorithms when handling dense spatial node configurations under non-rigid deformations, we conducted a multi-target identity verification benchmark using the forty-marker optimized spatial sampling layout. The recordings comprised the standard dynamic sequences (“Visemes” and “Emotions”), captured from a frontal viewpoint: both sequences of six participants and the “Emotions” sequence of the seventh, for thirteen multi-target sequences in total.

**Figure 8:**
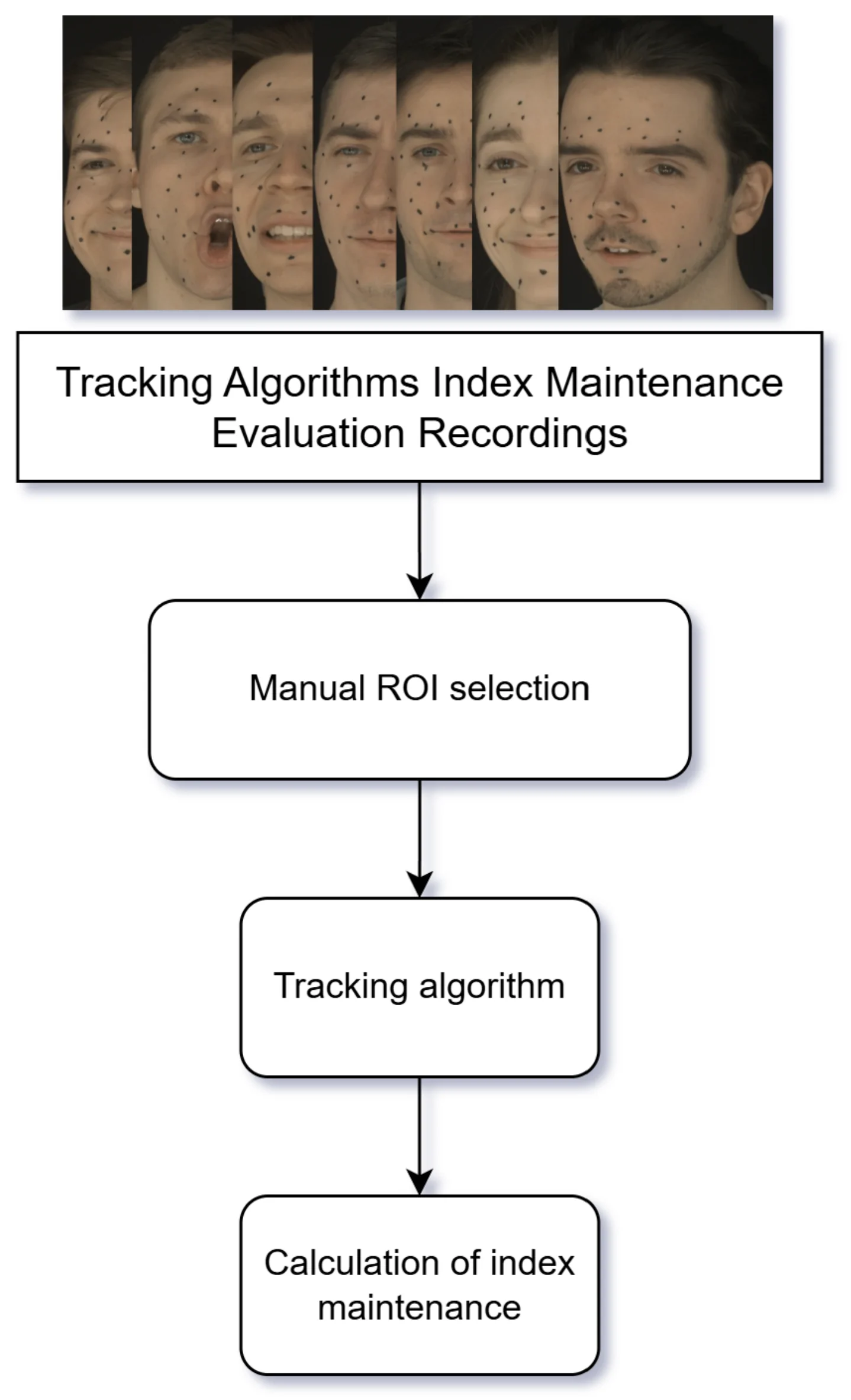
Evaluation protocol for correct marker index determination.

The tracking robustness was evaluated quantitatively as the percentage of correctly tracked markers remaining at the final frame of the sequence relative to the total number of initialized target nodes. For each marker, the target bounding box was initialized once in the first frame, establishing the initial state vector across all tracking algorithms. The evaluation protocol is schematically shown in Figure 8.

A final-frame percentage cannot distinguish between a marker lost early in a sequence and one lost near its end; therefore, two complementary measures are computed from the same per-frame retention data (Section 5.4): the normalised retention area (area under the correctly tracked count-versus-frame curve divided by the initial marker count and the sequence length, equal to 1 for a tracker that never loses a marker) and the median normalised survival time (fraction of the sequence elapsed when half the markers have been lost). The final-frame percentage is reported as the primary measure, as it is the most directly interpretable of the three.

Temporal tracking failure (loss of state maintenance) was defined under three conditions: (1) complete loss of the tracking target, where the algorithm stops updating; (2) spatial drift, where the tracker’s bounding box jumps to a neighboring marker (identity swapping); or (3) locking onto spurious background structures, such as skin folds or facial features. These instances were treated as tracking failures, and the corresponding nodes were classified as lost. In the per-frame data, condition (2) holds when the tracker’s centre is nearer to the reference position of another marker than to that of its own, and condition (3) holds when it lies more than two marker diameters from the reference position of its own marker. The initial boxes measure about three marker diameters; thus, beyond two diameters the marker is no longer inside the region that the tracker follows. A node was classified as lost once a failure had lasted ten consecutive frames (1/6 s); shorter interruptions, which include single frames in which the reference position itself is uncertain, were not counted as losses.

### 4.4. Computational Complexity and Latency Analysis

To evaluate the real-time operational capability and processing throughput of the eleven tracking algorithms, a computational complexity analysis was performed. The execution latency was measured frame-by-frame, isolating the core algorithmic execution time of the update() and bounding box retrieval methods. Auxiliary operations, such as video file I/O, frame decompression, and GUI rendering, were excluded from the timing loop.

All trackers were executed with OpenCV 4.10.0 built with the opencv_contrib modules on the default DNN backend and target (CPU); no GPU backend was used, and every tracker was constructed with its default parameters. The latency results reported in Section 5.5 were measured with the library restricted to a single thread by calling cv::setNumThreads(1) at start-up; because executing the application on a single thread does not by itself constrain the internal parallelism of OpenCV (parallel_for_) or the thread pool of the DNN module, this call is required for a single-thread comparison. With the default parallelism of the library on the same machine, Boosting, KCF, MOSSE, and ViT execute at the same speed, CSRT, MedianFlow, and Nano 1.5–1.7 times faster, GOTURN 1.9 times faster, DaSiamRPN 4.0 times faster, and MIL slightly slower; the reported latencies are therefore the single-thread cost, which constitutes an upper bound on the latency attainable in a multi-core deployment. The neural trackers used the following model files: GOTURN, goturn.prototxt and goturn.caffemodel from the opencv_extra repository; DaSiamRPN, dasiamrpn_model.onnx, dasiamrpn_ kernel_r1.onnx, and dasiamrpn_kernel_cls1.onnx; Nano, the NanoTrack v2 backbone and head networks [18]; and ViT, object_tracking_vittrack_2023sep.onnx from the OpenCV Zoo [20]. The hardware environment is given in Table 3.

## 5. Results

### 5.1. Baseline Reference Coordinate Verification

To quantify the agreement of the automated high-throughput Blob Detection pipeline with a sub-pixel extractor, its coordinate outputs were compared against the sub-pixel coordinates extracted by the Point Center Weighted Extractor (PCWE) control algorithm. Across five synchronized camera viewpoints during a continuous calibration sequence, the mean Euclidean distance between the centroids computed by the two algorithms was 0.28±0.24 pixels, which corresponds to approximately 0.07±0.06 mm at the nominal 0.24 mm/pixel image scale of the optoelectronic system used. In 3.5% of the marker frames, the two extractors located different blobs more than ten pixels apart; these frames are excluded from the values above.

This value characterises the agreement of the two extractors under controlled conditions, i.e., on rigid markers with well-defined boundaries and with both extractors sharing their coarse localisation stage. It indicates how reproducibly the reference centroid is located, but not whether it is biased, as both extractors share their coarse localisation stage; the deviations reported in Section 5.2 should be interpreted against an uncertainty band of at least this order.

On the hand-drawn markers during dynamic deformation (one frontal four-marker ’Emotions’ sequence), the two extractors agreed to 0.08±0.05 pixels (median 0.07, maximum 0.33 pixels); in 5.8% of the marker frames, they located different blobs more than ten pixels apart, and these frames are excluded. While this nominal deviation is lower than that observed for the rigid glued markers (0.28±0.24 pixels), this difference is due to the markers, not the viewpoint; in the same frontal view, the glued markers agreed to 0.28 pixels, presumably because the shading and highlights of the raised semi-spherical markers perturb the apparent intensity centroid whereas the drawn markers are flat. Consequently, the 0.08-pixel agreement confirms that dynamic tissue strain and local edge irregularity do not cause detector divergence under perpendicular viewing; however, it represents numerical consistency under ideal projection geometry.

On the 668 marker frames in which both annotators marked the marker, the Blob Detection reference deviated from the manual centres by 0.52 pixels on average (approximately 0.12 mm; median 0.43 pixels, 95th percentile 1.09 pixels; 0.45 and 0.64 pixels in the four-marker and forty-marker sequences), with a mean offset (reference minus manual centre, *y* pointing down the image) of +0.04 pixels along *x* and +0.20 pixels along *y* and 95% limits of agreement of −0.83 to +0.91 and −0.75 to +1.16 pixels. The annotators deviated from each other by 0.64 pixels and from their own repeated marks by 0.52 and 0.70 pixels, so the reference is at least as reproducible as manual annotation; it returned no position in 2.2% of these marker frames. The mean distance of each tracker from the manual centres differed from its distance from the reference by at most 0.13 pixels, and both distances ordered the eleven trackers and the hybrid pipeline (Section 6.1) almost identically (Kendall’s τ=0.97), so the ordering of the trackers does not depend on the choice of reference.

**Figure 9:**
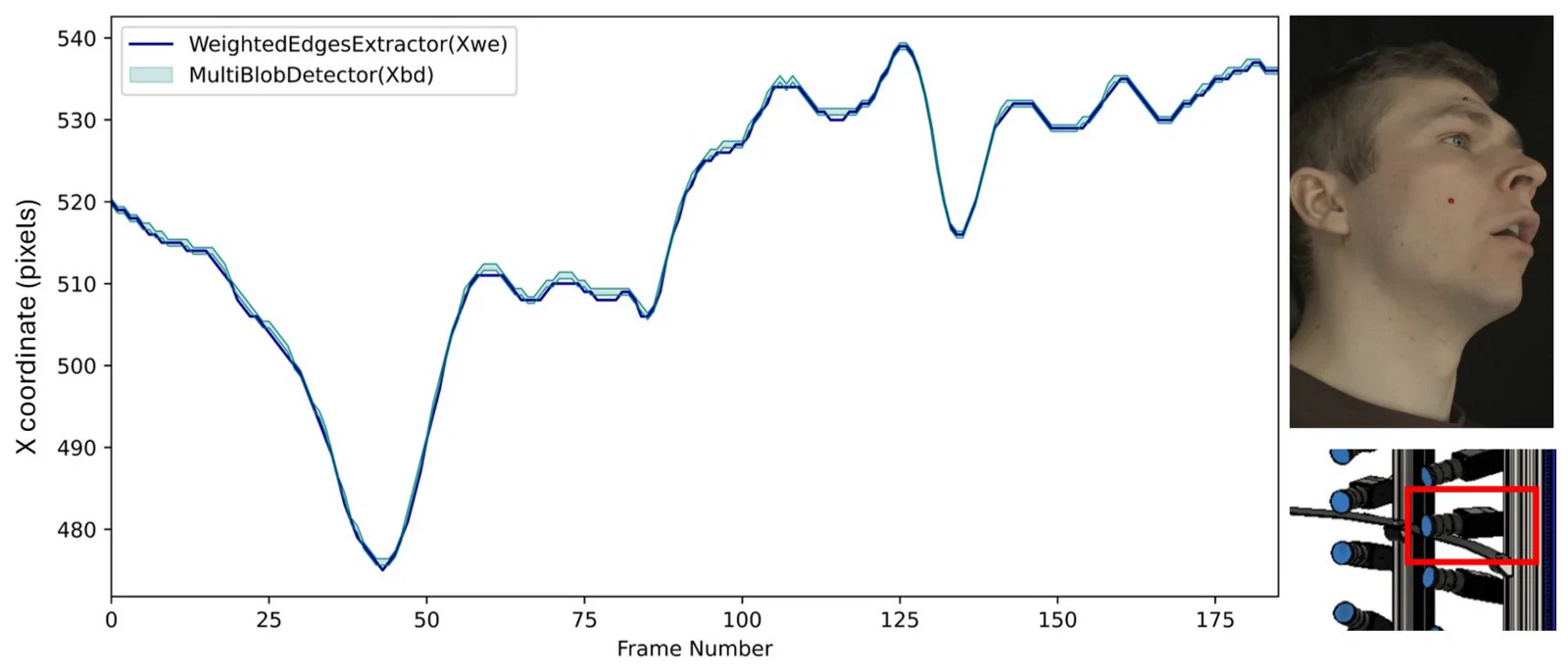
Comparison of $X_{\mathrm{BD}}$-coordinate (Blob Detector) and $X_{\mathrm{WE}}$-coordinate (Point Centre Weighted Extractor) for a selected marker and camera view. The red rectangle marks the camera whose recording is plotted.

Figure 9 displays the temporal alignment of the computed $X_{\mathrm{BD}}$ (Blob Detection) and $X_{\mathrm{WE}}$ (PCWE) coordinates for a representative passive epidermal marker.

### 5.2. Deviation from the Reference

The deviations from the reference, calculated over 223 marker trajectories (seven subjects, two motion sequences, five camera viewpoints, and up to four markers visible from each viewpoint), each tracked by the eleven algorithms (2453 tracker–trajectory pairs), are summarized in Table 4. The absolute component deviations in the horizontal ($d_{x}$) and vertical ($d_{y}$) directions are reported alongside the Euclidean distances, in pixels and approximate millimetres.

**Figure 10:**
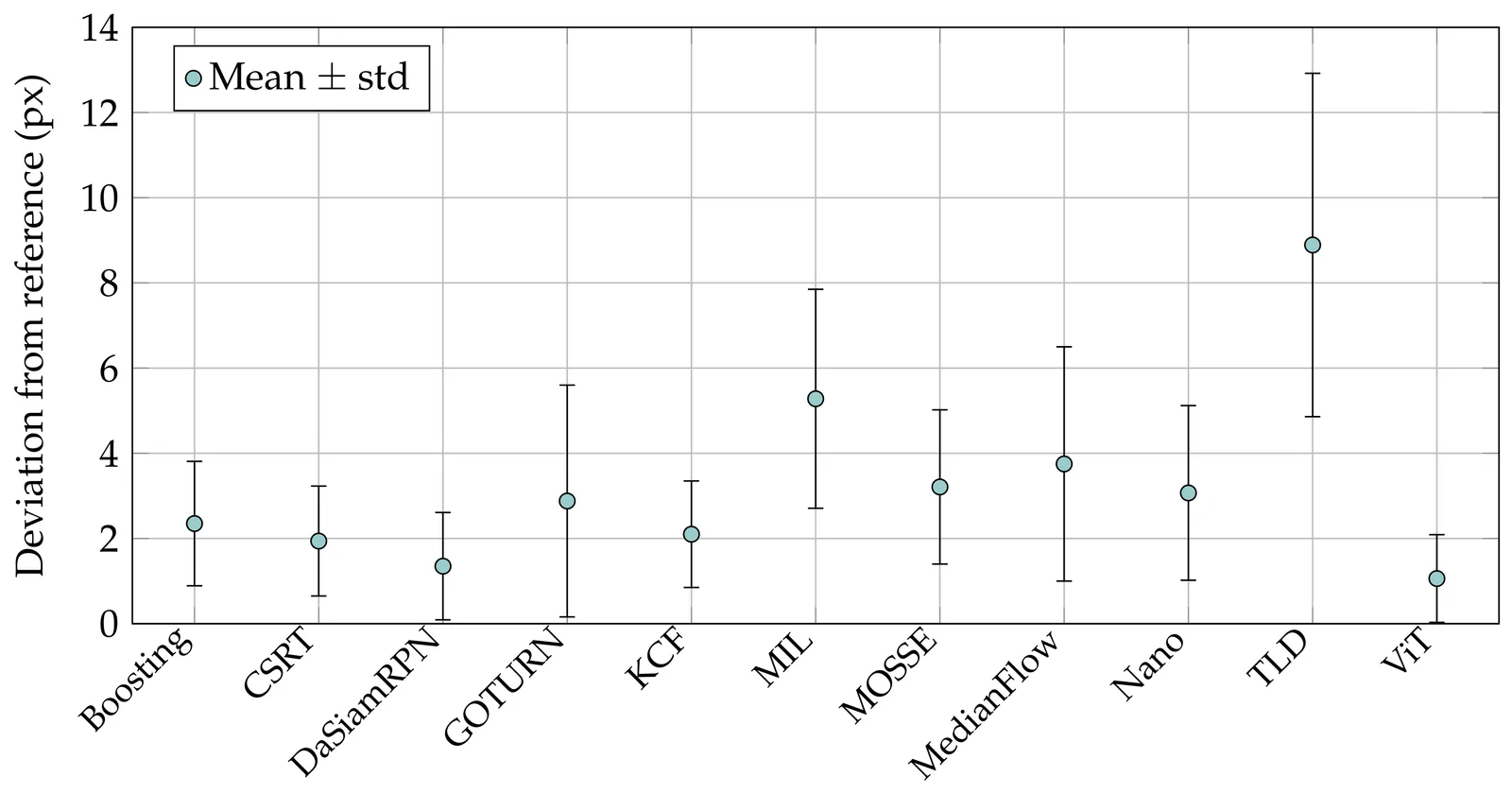
Mean deviation from the reference per tracker with the pooled per-frame standard deviation (filled markers, mean±std) from Table 4; the maxima, which are bounded by the failure criterion, are listed in the table. The pooled standard deviation describes the spread of correlated per-frame observations, not the uncertainty of the mean; subject-level intervals are given in the text.

To visualize the deviation distribution per tracker, Figure 10 presents the mean deviation from the reference of each tracker with its standard deviation.

As detailed in Table 4, the smallest mean deviations from the reference were recorded for the neural network-based trackers ViT (1.06±1.03 pixels, approximately 0.25±0.25 mm) and DaSiamRPN (1.35±1.26 pixels, approximately 0.32±0.30 mm). Among the classical algorithms, KCF and CSRT showed the smallest deviations, with mean Euclidean distances of 2.10±1.25 pixels (0.50±0.30 mm) and 1.94±1.29 pixels (0.46±0.31 mm), respectively. The largest deviations were recorded for the TLD (8.89±4.03 pixels) and MIL (5.28±2.57 pixels) trackers. These metrics represent pixel-level deviations computed only when the tracking state was active, excluding frames where the target was lost; the fraction of frames on which each tracker remained active is listed in the last column of Table 4. It lies between 97 and 99% for most trackers, but is 84.5% for KCF, 82.0% for TLD, 68.1% for GOTURN, and 49.2% for MOSSE, indicating that their deviations describe a smaller share of the frames.

The four leading trackers are ViT, DaSiamRPN, CSRT, and KCF, which span 1.06 to 2.10 pixels with standard deviations of 1.03 to 1.29 pixels; thus, their distributions overlap substantially. They are reported here as a single descriptive leading tier, with MedianFlow, Boosting, MOSSE, Nano, and GOTURN forming an intermediate tier and MIL and TLD a trailing tier. A percentile bootstrap over subjects (n=7; per-trajectory means of the frontal-view four-marker trajectories, aggregated to one value per subject, 10,000 resamples) gives approximate 95% intervals of the mean deviation of 0.93–1.36 pixels for ViT, 1.14–1.49 pixels for DaSiamRPN, 1.67–2.42 pixels for CSRT, and 1.83–2.75 pixels for KCF, whereas the intervals of MIL (5.36–6.46 pixels) and TLD (9.80–12.60 pixels) lie entirely above them. The subjects ranked the eleven trackers consistently (Friedman $\chi^{2}=62.5$, ten degrees of freedom, $p<10^{-8}$; Kendall’s W=0.89). Within the leading tier, ViT, and DaSiamRPN deviated less than CSRT and KCF in at least six of the seven subjects, and the paired bootstrap intervals of these four differences exclude zero (for example, ViT minus CSRT: −0.95 pixels, 95% interval −1.28 to −0.63 pixels). With seven subjects, however, the smallest attainable two-sided *p*-value of the exact paired sign-flip test is 0.016, so after Holm correction across the thirteen pairs, no difference can reach significance (adjusted p≥0.20); therefore, the differences within the leading tier are indicative but not confirmed.

**Figure 11:**
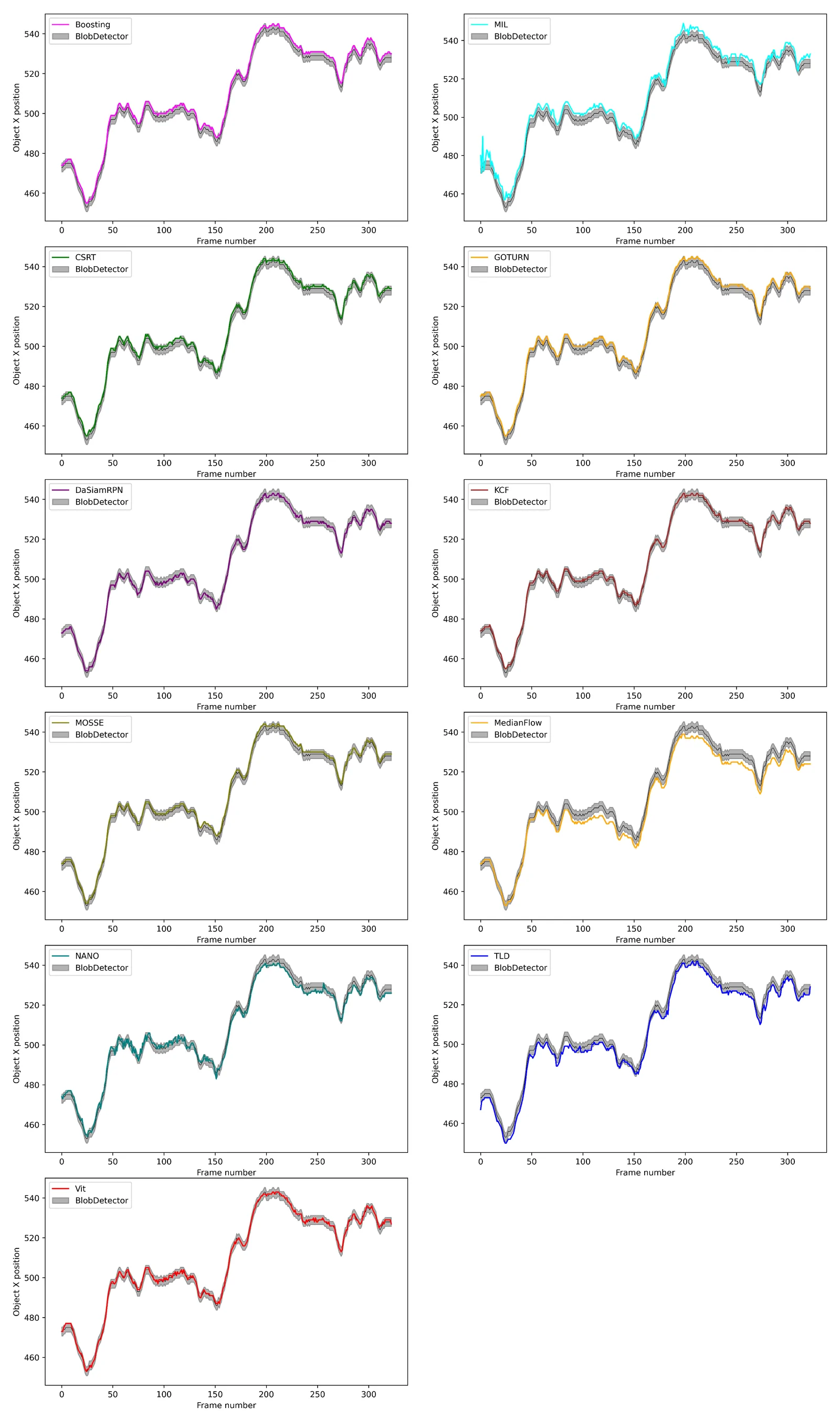
Computed *X*-coordinate trajectories of the evaluated tracking algorithms relative to the Blob Detection reference, shown as the black line within the shaded band.

An example plot displaying the computed and reference *X*-coordinates for a selected epidermal marker across a single frontal camera sequence is illustrated in Figure 11. The reference trajectory is indicated by the central black line within the shaded grey region.

**Figure 12:**
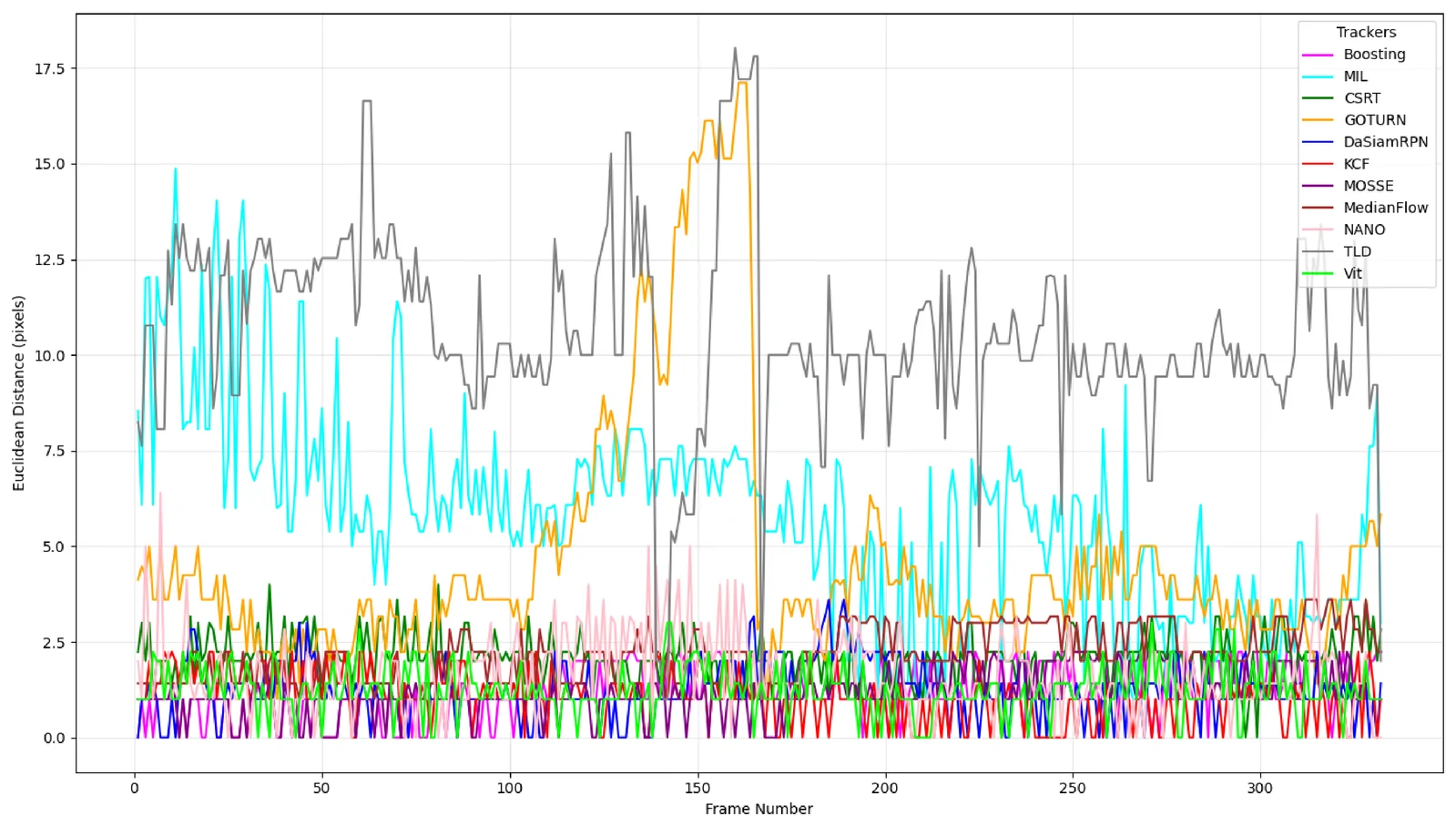
Deviation from the reference over the frames of one sequence for a single tracked marker and for all eleven trackers.

Figure 12 shows the instantaneous Euclidean deviation from the reference across the frames of a representative dynamic sequence for all eleven trackers, making the separation between the leading, intermediate, and trailing tiers visible over time.

### 5.3. Sensitivity to the Initial Region

The influence of the initial region size was examined on the fourteen frontal four-marker sequences by rebuilding each initial region with a side of *s* times the diameter of its marker for s∈{1.5,2.0,2.5,3.0} (median box sides of 20, 26, 32, and 39 pixels) and running all eleven trackers from identical regions at each scale. Table 5 reports the mean deviation conditional on active tracking and the fraction of frames on which each tracker remained active at each scale.

Two effects are apparent. First, the correlation filter trackers KCF and MOSSE require a region that is considerably larger than the marker: at s=1.5, MOSSE remained active on only 5% of the frames and KCF on 57%, whereas at s≥2.5 both remained active on most of the frames. Therefore, their low conditional deviations at small *s* are computed over a small surviving subset of frames, which is made visible by the active-fraction column. Second, among the trackers that remained active throughout, the deviation increases with the region size for Nano, MedianFlow, and MIL (from 1.6, 3.1, and 3.8 pixels at s=1.5 to 3.6, 4.0, and 5.9 pixels at s=3.0), does so slightly for DaSiamRPN (from 1.1 to 1.3 pixels), and is nearly constant for ViT (1.1 pixels) and CSRT (2.1–2.3 pixels). Boosting remained active on 89% of the frames at s=1.5 and on 97–98% at the larger scales, with a nearly constant deviation. GOTURN and TLD lost their markers in a substantial share of the frames at every scale. The manually defined boxes used for Table 4 measured approximately three times the marker diameter, corresponding to s≈3. ViT and DaSiamRPN show the smallest deviations at every scale, whereas Nano depends most on the region size; with regions of s≤2, it deviates less than CSRT and KCF. Therefore, the values in Table 4 are specific to that region size.

### 5.4. Results of Temporal State Maintenance and Multi-Target Identity Verification

**Figure 13:**
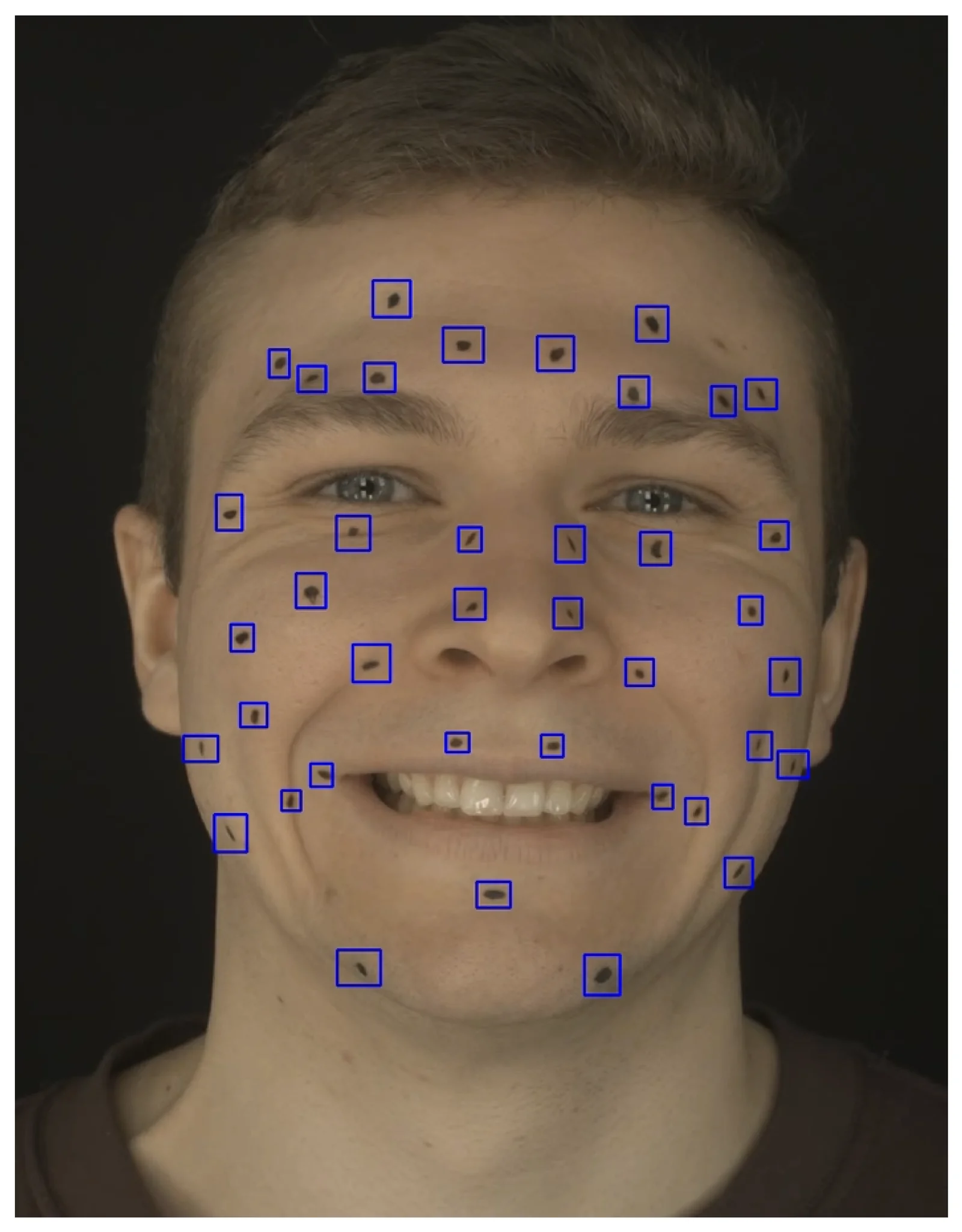
Simultaneous multi-target tracking visualization showing initialized regions of interest for the forty-marker spatial layout.

The temporal robustness of the tracking state was evaluated across thirteen dynamic multi-target sequences of the seven participants featuring the mathematically optimized forty-marker spatial sampling layout. Figure 13 illustrates an exemplary frame of the CSRT algorithm tracking all markers simultaneously.

**Figure 14:**
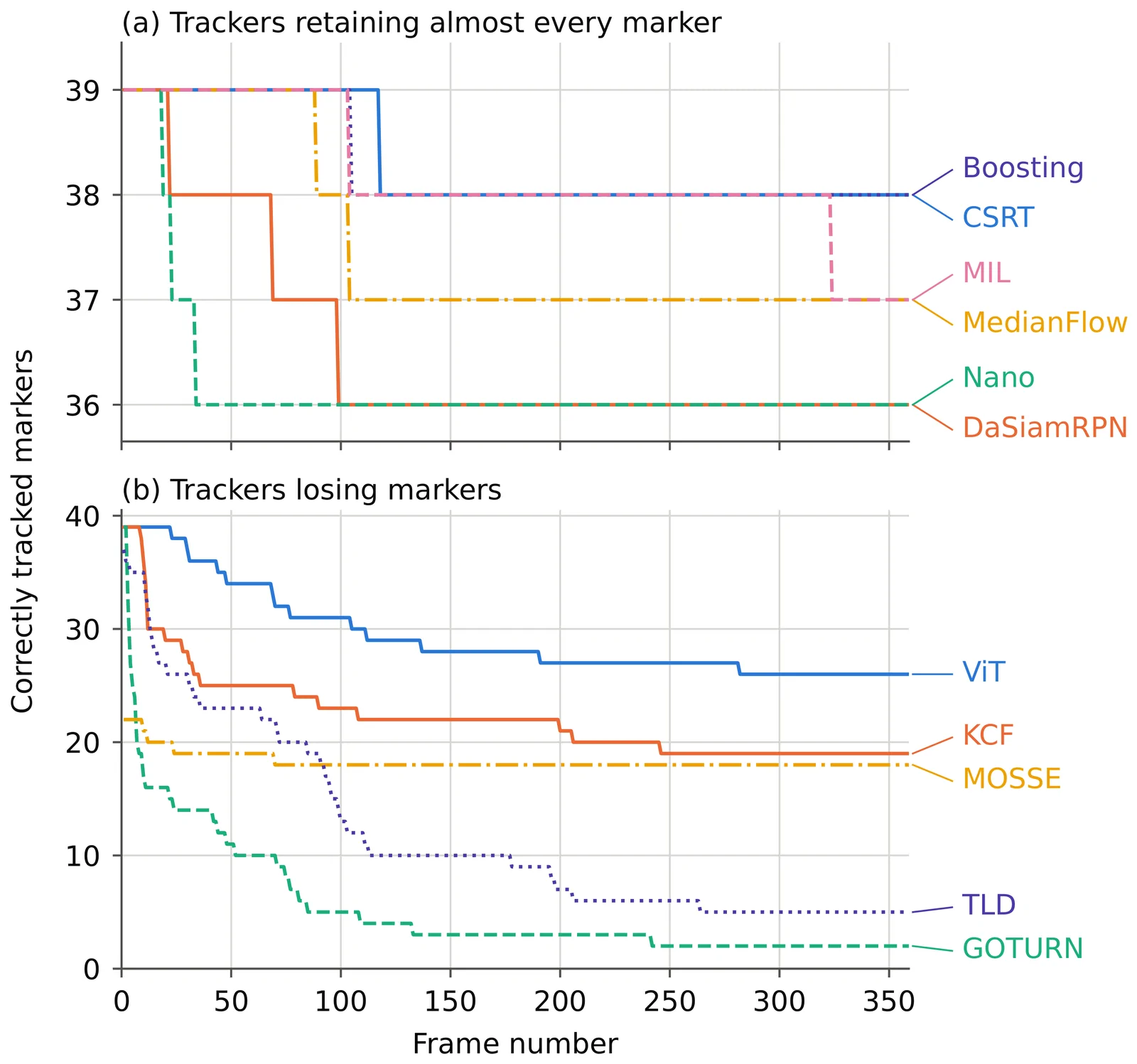
Temporal retention of correctly tracked markers across the frames of one representative forty-marker sequence: (**a**) the six trackers that retain almost every marker, on an expanded vertical scale; (**b**) the five trackers that lose markers, on the full scale. The curves coincide wherever two trackers hold the same number of markers; MOSSE acquired only 22 of the 39 markers at initialisation.

The active tracking state of each node was monitored continuously over the sequences, with tracking failures categorized into complete target loss, spatial bounding box drift (locking onto adjacent skin features), or identity swapping (index confusion) between neighboring nodes. Figure 14 shows the number of active correctly indexed tracking targets across frames for a single representative sequence.

**Figure 15:**
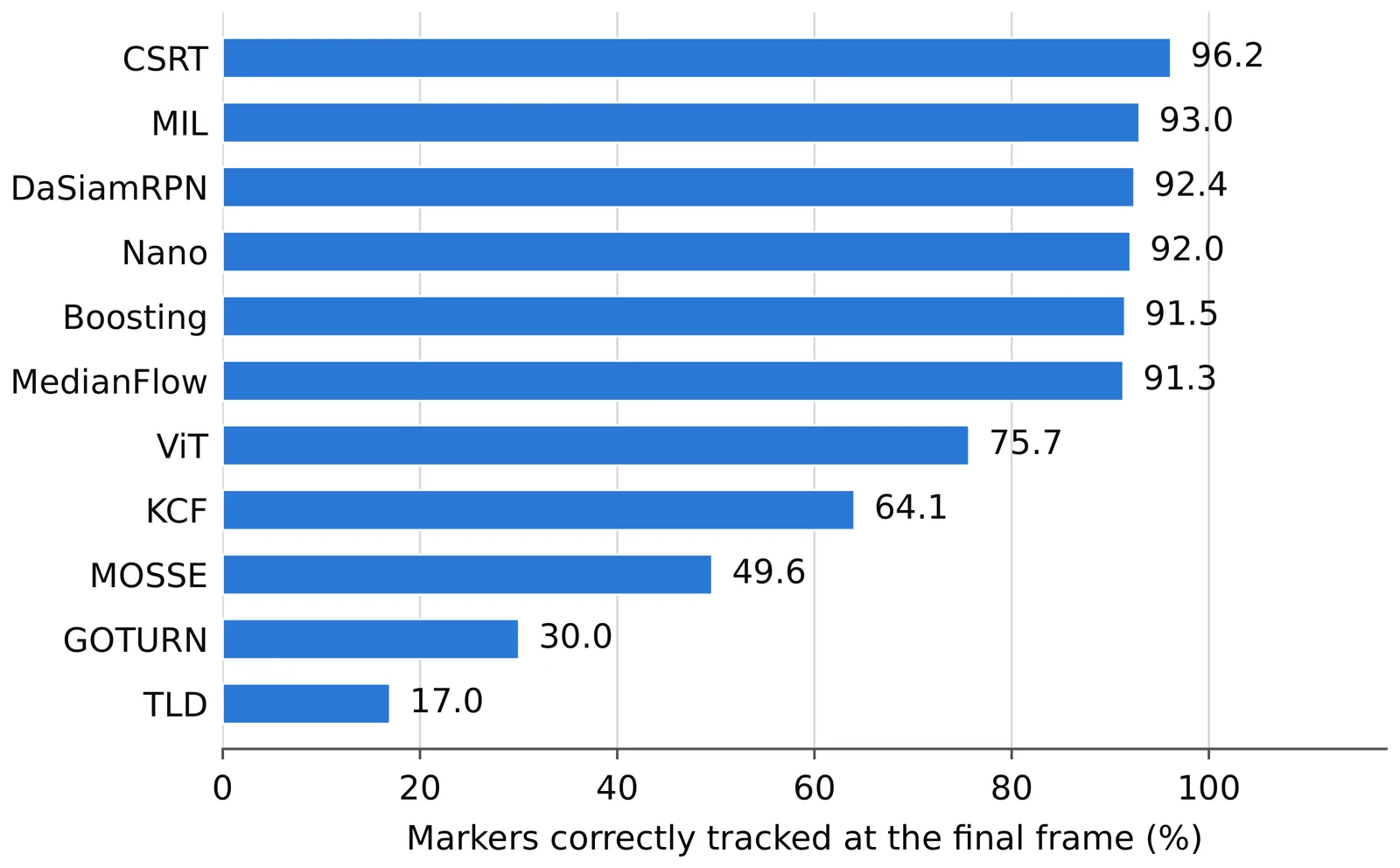
Percentage of passive epidermal markers still correctly tracked and indexed at the final sequence frame, relative to the initial target nodes, as a mean over the seven participants.

The final tracking success rate, computed as the ratio of correctly tracked and indexed markers at the final frame of the sequence relative to the initial target nodes, is shown in Figure 15 as a mean over the seven participants.

The two failure timing measures of Section 4.3 were computed from the per-frame data of Figure 14. For the six trackers that retain more than 80% of the markers, the normalised retention area lies between 93 and 98% and more than half of the markers survive to the final frame, so neither measure adds to the final-frame percentage. The two measures separate the five trackers that lose markers. ViT keeps more than half of its markers to the final frame, with a retention area of 75%. GOTURN, which retains 5.1% of the markers at the final frame of this sequence, has the lowest retention area of all eleven trackers at 14%, because its losses occur within the first 2% of the sequence. MOSSE acquired only 22 of the 39 markers at initialisation and had lost half of them after 6% of the sequence, with a retention area of 47%; KCF, with similar final-frame retention (48.7% against 46.2%), reaches a retention area of 57% and loses half of its markers after 68% of the sequence; TLD, with a final-frame retention of 12.8%, has a retention area of 30% and a median survival time of 23%. Therefore, the final-frame percentage does not distinguish the early losses of MOSSE from the late losses of KCF.

**Figure 16:**
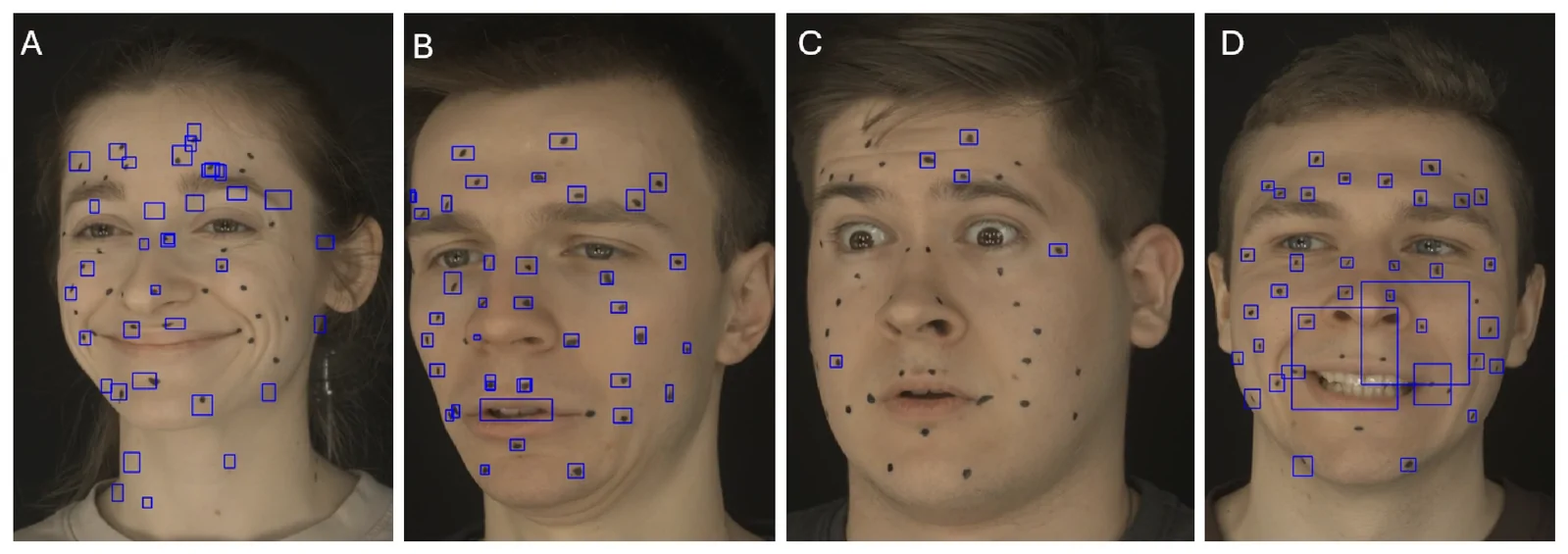
Observed tracking failure modes, including (**A**) TLD target loss, (**B**) Nano feature switching, (**C**) KCF occlusion failure, and (**D**) MedianFlow bounding box expansion.

During the temporal evaluations, several distinct failure modes were observed:

- TLD: Exhibited rapid state maintenance failures, losing or misidentifying the majority of active nodes shortly after initialization (Figure 16A).
- MOSSE and KCF: The MOSSE tracker frequently failed to initialize tracking targets. Both MOSSE and KCF failed to recover active tracking states after transient self-occlusions (e.g., skin folds or rapid muscle contractions), resulting in permanent target loss (Figure 16C).
- Boosting: Successfully maintained active tracking states but occasionally exhibited identity swapping (index confusion) when target nodes passed in close physical proximity, leading to permanent misidentification. Additionally, it exhibited high-frequency spatial jitter during dynamic motion.
- MedianFlow: Upon encountering localized occlusions, the tracking bounding boxes expanded significantly, causing the tracker to drift and lock onto adjacent skin features or unrelated facial structures (Figure 16D).
- MIL: Maintained a high tracking success rate, but exhibited high-frequency spatial jitter similar to the Boosting tracker.
- CSRT: Achieved the highest tracking success rate among classical trackers, maintaining correct indexing for 96.2% of target nodes with minimal spatial jitter.
- GOTURN: Frequently drifted from the designated marker, locking onto high-contrast facial structures such as the eyes or the mouth, resulting in a final success rate of 30.0%.
- Nano & ViT: Exhibited occasional bounding box drift and feature switching (e.g., re-locking from epidermal markers to the lip boundary, Figure 16B), resulting in success rates of 92.0% and 75.7%, respectively.
- DaSiamRPN: Demonstrated the highest temporal stability among the neural network-based trackers, successfully tracking 92.4% of target nodes with identity swaps in only 1.6% of its active frames.

**Figure 17:**
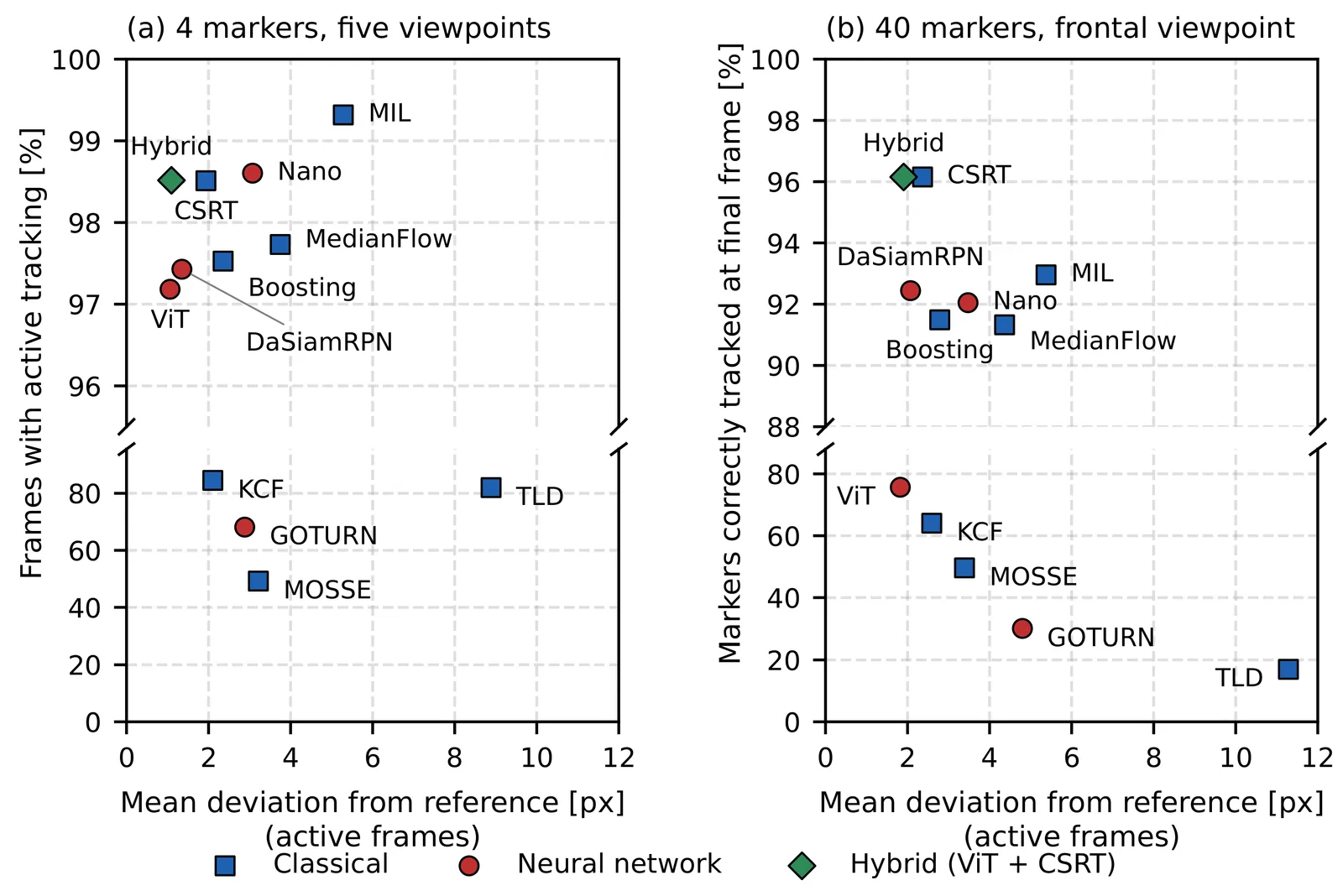
Tracking stability versus deviation from the reference for all evaluated trackers and the hybrid pipeline, with both axes of each panel measured on the same sequences: (**a**) four-marker configuration, five viewpoints, share of frames with active tracking; (**b**) forty-marker configuration, frontal viewpoint, markers correctly tracked at the final frame. Deviations are means over active frames; the upper part of each vertical axis is expanded.

Figure 17 relates the deviation from the reference to tracking stability separately for the two configurations, with both axes of each panel measured on the same sequences: the share of frames with active tracking for the four-marker sequences, and the final tracking success rate for the forty-marker sequences. The two configurations rank the trackers almost identically by deviation (Kendall’s τ=0.85 between the mean deviations of the eleven trackers); only GOTURN deviates relatively more in the dense layout.

### 5.5. Computational Latency and Throughput Analysis

**Figure 18:**
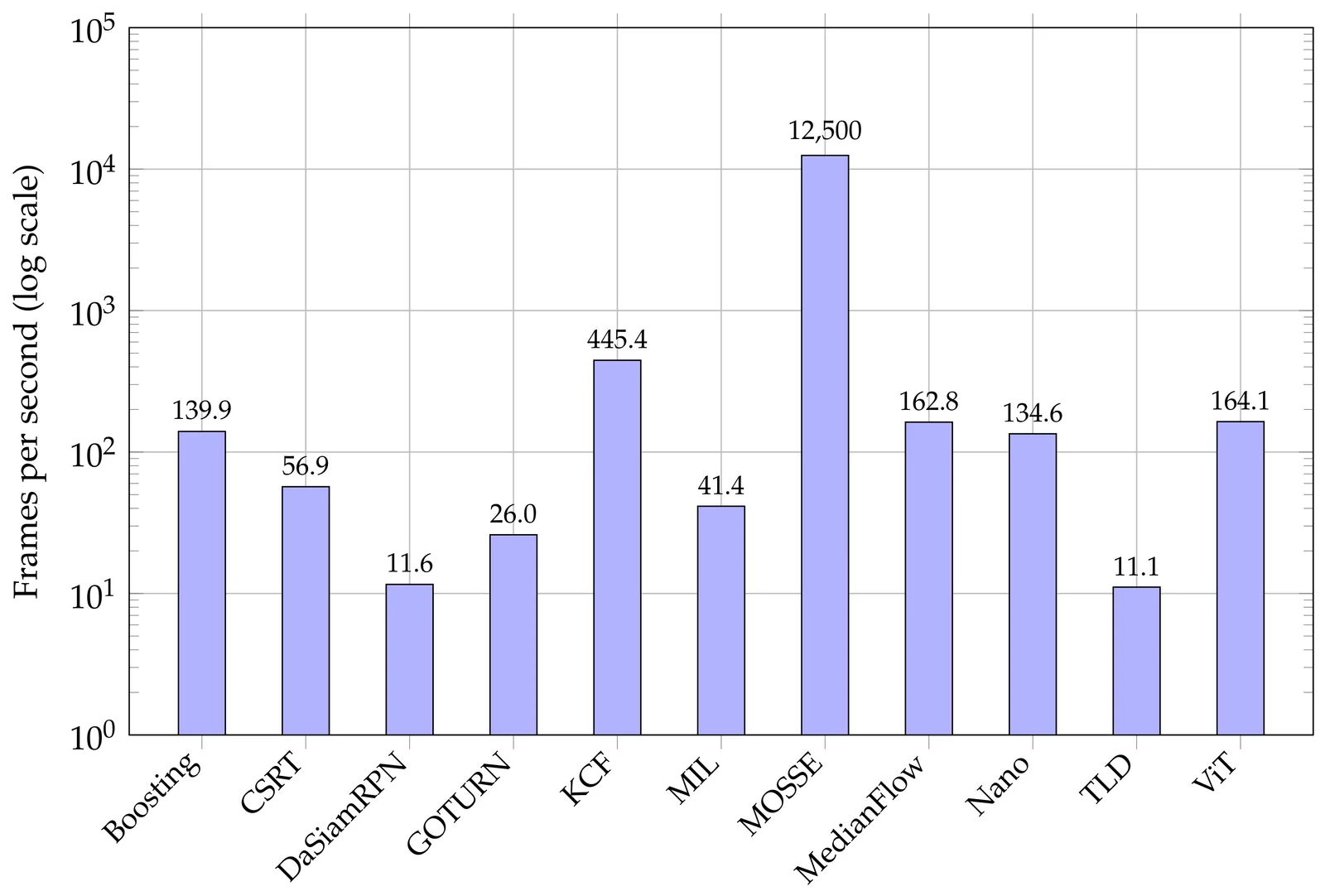
Algorithmic throughput in frames per second (logarithmic scale), from Table 6; single thread.

Table 6 presents the computational latency of the tracking algorithms, measured frame-by-frame as the execution time of the tracking update and coordinate retrieval operations. The processing throughput is expressed in frames per second (FPS) on a logarithmic scale in Figure 18.

## 6. Discussion and Future Works

The comprehensive evaluation of the eleven tracking algorithms conducted in this study provides practical insights into the deployment of passive epidermal coordinate acquisition pipelines on deforming non-rigid surfaces. Although tracking high-contrast dark markers on relatively bright skin might initially appear to be a simplified computer vision task, the highly nonlinear viscoelastic deformations of human facial tissue during emotional expression and phonetic articulation introduce severe tracking challenges. These challenges are characterized by rapid transient self-occlusions, localized skin folding, dynamic self-shadowing, and the spatial proximity of adjacent markers, which collectively test the physical and mathematical limits of trackers. By addressing these phenomena under controlled optoelectronic conditions, this study establishes a robust and reproducible benchmark for future sensor pipelines.

In the following, deviations refer to the four-marker sequences (five viewpoints) and retention to the forty-marker sequences (frontal viewpoint); the two are measured on different sequences, and as such are discussed side-by-side without combining them into one score. Among the classical trackers evaluated, the Channel and Spatial Reliability Tracker (CSRT) was the most robust choice for dynamic multi-target coordinate acquisition under the conditions of this study. Results showing a tracking success rate of 96.2% at the end of the dynamic sequences while maintaining a low mean Euclidean deviation of 1.94±1.29 pixels (0.46±0.31 mm) reflect the stability of CSRT’s formulation. The integration of spatial and channel reliability maps dynamically restricts the filter’s spatial support to the most stable regions of the marker, preventing the bounding box from drifting onto adjacent transient skin folds or micro-textures. While the Kernelized Correlation Filter (KCF) matched CSRT’s deviation (2.10±1.25 pixels, or 0.50±0.30 mm) at a much higher execution throughput of 445.4 FPS, its mathematical formulation lacks long-term memory and spatiotemporal constraints; consequently, KCF is highly vulnerable to transient self-occlusions and a single frame of marker concealment (e.g., during deep tissue folds in the “Shocked” or “Angry” sequences) can lead to irreversible tracking failure, reducing its global tracking success rate to just 64.1%.

This behavior highlights a critical finding: although CSRT and KCF are consistently ranked as the top classical trackers in short-duration or rigid-body tracking challenges (such as liver tumor tracking in ultrasound sequences, where occlusion is rare [12]), their performance profiles diverge drastically in high-density non-rigid surface sensing scenarios where occlusions and deformations are dominant. This shows that per-frame deviation alone is an insufficient metric for evaluating optoelectronic sensor pipelines; long-term temporal stability and occlusion recovery mechanisms must be treated as co-primary performance indicators.

Among the neural network-based trackers, the Vision Transformer-based tracker (ViT) recorded the smallest mean deviation of all evaluated algorithms (within a leading tier where differences were not significant at the subject level, Section 5.2), yielding a mean Euclidean deviation of 1.06±1.03 pixels (approximately 0.25±0.25 mm) at a high computational throughput of 164.1 FPS. This low deviation is consistent with ViT’s multi-head self-attention mechanism which computes global dependencies across image patches, allowing the bounding box to adapt dynamically to the non-rigid morphological changes of the drawn markers. However, this global attention mechanism lacks strong local spatiotemporal constraints, making ViT susceptible to feature switching or background drift. Under extreme localized strain, such as the rapid jaw and lip movements in the “Visemes” sequence, the tracker frequently locks onto neighboring high-contrast epidermal structures, e.g., the lip boundary or deep facial furrows, resulting in a lower final success rate of 75.7%.

Conversely, the Distractor-Aware Siamese Region Proposal Network (DaSiamRPN) proved to be the most stable neural network-based tracker, maintaining correct indexing for 92.4% of target nodes with a mean deviation of 1.35±1.26 pixels (approximately 0.32±0.30 mm). By incorporating semantic background information during offline training and dynamically updating its template model to actively suppress similar-looking adjacent features, DaSiamRPN showed little identity swapping or feature switching in these sequences (1.6% of its active frames). This made DaSiamRPN the most reliable of the four OpenCV deep learning trackers evaluated for high-density long-duration tracking sequences where coordinate identity maintenance is paramount. However, its single-thread execution latency of 85.90 ms (yielding 11.6 FPS) represents a bottleneck for real-time low-latency deployments. When comparing ViT to Nano, the latter being a lightweight Siamese architecture, ViT showed lower deviation and higher throughput, while Nano achieved a higher final tracking stability of 92.0% due to its more constrained local search space.

The performance of classical and lightweight deep trackers also revealed significant limitations under dynamic conditions. The TLD tracker experienced catastrophic failures across almost all sequences, resulting in a final success rate of only 17.0%. TLD’s parallelized tracking, learning, and detection architecture frequently generates conflicting bounding box hypotheses on dense identical targets, causing the tracker to oscillate, lose target lock, or merge multiple trajectories. Similarly, MedianFlow, despite its high throughput of 162.8 FPS and final retention of 91.3%, exhibited a severe bounding box expansion failure mode. Upon encountering transient self-occlusions, the forward–backward error estimation fails, causing the bounding box to grow exponentially and lock onto unrelated high-contrast facial structures. The Boosting and MIL trackers, while retaining most markers (91.5% and 93.0% success, respectively), exhibited high-frequency spatial jitter during dynamic sequences, which introduced artificial high-frequency noise into the computed trajectories and reduced their suitability for high-fidelity coordinate acquisition. Furthermore, MIL showed a high deviation of 5.28±2.57 pixels (approximately 1.27±0.62 mm) among the trackers that produced stable tracking.

The Minimum Output Sum of Squared Error (MOSSE) tracker exhibited an extraordinary computational throughput of about 12,500 FPS, outperforming all other algorithms by several orders of magnitude. This throughput is achieved by computing adaptive correlation filters in the Fourier domain using the Fast Fourier Transform (FFT), reducing the computational complexity of the coordinate update to element-wise multiplications. However, this simplicity comes at a severe cost to robustness: MOSSE achieved a global tracking success rate of only 49.6%. It struggled heavily during the initialization phase, frequently failing to lock onto hand-drawn markers with minor morphological imperfections, and was entirely unable to recover from even momentary occlusions.

Importantly, all latency and throughput measurements reported in this benchmark were obtained on the CPU of an Intel Core i9-13900H processor with OpenCV pinned to a single thread and with no GPU acceleration (Section 4.4). This was a methodological decision to ensure a fair and hardware-agnostic comparison. Enabling GPU acceleration would introduce external hardware-dependent sources of latency such as PCI–express bus transfer times and thread scheduling overhead that would obscure the pure algorithmic and computational complexity of the underlying mathematical frameworks. In a production deployment, deep learning models (DaSiamRPN, ViT, GOTURN, and Nano) are typically executed on dedicated GPU or TPU hardware, where their processing latency is expected to drop by an order of magnitude. Under such conditions, these neural network-based trackers would exceed real-time processing thresholds, combining their low deviation with high computational throughput. Future work should extend this benchmark to dedicated GPU architectures in order to evaluate the hardware-scaled performance of these algorithms.

The component analysis showed no consistent difference between the horizontal (*x*) and vertical (*y*) deviations: the horizontal component was the larger for six of the eleven trackers, but by small margins except for GOTURN (Table 4). The neural network-based trackers demonstrated a superior ability to dynamically adapt the aspect ratio and scale of their bounding boxes to the deformations of the markers; conversely, classical correlation filter-based trackers exhibited high geometric rigidity, maintaining fixed bounding box dimensions that led to larger deviations from the reference as the physical marker deformed underneath. However, this adaptability of neural trackers is a double-edged sword: if the initial user-defined ROI is too large, the dynamic bounding box is more likely to expand and capture adjacent high-contrast facial features, initiating a drift or feature switching failure.

A notable observation of this study is the consistency of tracker behavior across all seven human subjects. Despite variations in skin reflectance, tissue elasticity, and individual facial geometry, the relative performance ranking, failure modes, and deviation distributions of the eleven tracking algorithms remained qualitatively consistent across participants. The cohort is homogeneous (Section 6.4), so this consistency is established within it, and a wider cohort would be needed to extend it to other populations. Similarly, the trackers were ordered almost identically by deviation in the two marker layouts (Kendall’s τ=0.85), although the deviations were larger in the dense layout, where adjacent markers are in close proximity (e.g., near the lips during the “Visemes” sequence). Under these high-density conditions, trackers lacking spatial reliability maps (most notably GOTURN and TLD) frequently suffered from index confusion and trajectory merging.

Furthermore, the results indicate that a tracker’s active-frame deviation does not necessarily correlate with its long-term stability in state maintenance. For example, the MOSSE tracker, though failing to track 50.4% of the markers across full sequences, maintained a relatively low spatial coordinate deviation in the limited frames where it did successfully maintain target lock. This decoupling underscores the necessity of a multi-dimensional benchmarking protocol that separately evaluates initialization success, active-frame deviation, and continuous state maintenance robustness. To expand the utility of this work, future research should evaluate these tracking algorithms on low-power resource-constrained embedded platforms such as the Raspberry Pi or specialized edge AI compute modules. Lightweight classical algorithms such as KCF or MedianFlow and highly optimized deep learning models like TrackerNano represent promising candidates for low-cost portable optoelectronic coordinate acquisition systems that operate without high-end desktop CPUs or GPUs.

One scoping limitation applies to the results above: the retention results were obtained from the frontal viewpoint, and retention at oblique viewpoints still remains to be measured.

### 6.1. Cooperative Hybrid Pipeline

The complementary performance profiles identified in this study suggest that a strong practical solution for high-density non-rigid optoelectronic sensing may lie in a cooperative hybrid tracking pipeline. Rather than deploying a single algorithm, a hybrid framework could employ the Vision Transformer-based tracker (ViT) as the primary tracker in order to exploit its low deviation (approximately 0.25 mm) and high execution speed (164.1 FPS); concurrently, a robust tracker like CSRT or DaSiamRPN could run alongside it to monitor tracking confidence, detect bounding box drift, and act as a fallback state corrector. Upon detecting a tracking anomaly or localized occlusion, the system would temporarily switch to the fallback tracker in order to re-localize the marker, correct its index in time, and re-initialize the ViT search window. The switching criterion, re-initialisation procedure, and expected cost are as follows: the fallback advances on every frame alongside the primary; while the centres of the two trackers lie within about one marker diameter of each other (a third of the shorter side of the initial box), the position of the primary is used, since its deviation from the reference is the smaller. When the primary reports failure or the two disagree by more than this distance, the position of the fallback is used for that frame. The primary is re-initialised from the fallback’s box when it reports failure or after ten consecutive frames of disagreement; a re-initialised primary takes its template from the fallback’s box and thereby inherits the fallback’s centring deviation, so a brief disagreement only hands over the frame. Because both trackers advance every frame, the cost is approximately the sum of the two per-frame latencies.

This pipeline was implemented with ViT as the primary tracker and CSRT as the fallback, then evaluated in both configurations against each component running alone. The three pipelines were compared on the forty-marker sequences in Table 7. The position of the fallback was used in 4.5% of target frames, ranging from 1.9% to 9.4% per sequence, and the measured per-frame cost matched the sum of the two components to within 1%.

The hybrid retained as many markers as CSRT, equalling or exceeding it in every sequence, and reduced the mis-assignment rate of ViT to that of CSRT, so it recovers the identity losses of the primary tracker. At 1.90 pixels, its deviation is close to that of ViT (1.82 pixels) and lower than that of CSRT (2.37 pixels), and was lower than that of CSRT in eleven of the thirteen sequences; this is because the position of the primary is used whenever the two trackers agree. On the four-marker sequences of Table 4, the hybrid deviated by 1.09±1.05 pixels, against 1.94±1.29 pixels for CSRT and 1.06±1.03 pixels for ViT, and was active on 98.5% of the frames, as CSRT was. Thus, the hybrid combines the retention of CSRT with a deviation close to that of ViT, though at the cost of running both trackers.

The difference between the deviation of ViT alone in Table 4 (1.06±1.03 pixels) and in Table 7 (1.82 pixels) reflects the marker density: Table 4 measures centring on well-separated markers, whereas in the dense layout ViT more often drifts towards neighbouring structures before it is counted as lost. Tuning the switching criteria on held-out sequences remains future work.

### 6.2. Relation to Tracking Benchmarks and Facial Datasets

Table 8 situates this dataset against the established single-object tracking benchmarks, while Table 9 situates it against public facial capture and facial landmark tracking datasets. The distinguishing properties are target density, target similarity, and non-rigid deformation: general benchmarks overwhelmingly track a single visually distinctive object, whereas the present task tracks up to forty near-identical achromatic targets that deform independently. Except for IEMOCAP, in which 53 facial markers were reconstructed in 3D by an optical motion capture system, the facial datasets record unmarked faces as 3D scans or video and describe them using 3D meshes or landmarks.

In Table 8, occlusion is given as the share of marker frames in which the reference located the marker, an upper bound for the occluded share because the reference can also miss a visible marker, and deformation as the relative change in distance between neighbouring markers over the edges of a Delaunay triangulation of their initial positions, which excludes in-plane head motion. The other datasets of Table 8 and Table 9 report occlusion, if at all, as an annotated attribute, or for IEMOCAP, as missing frames per marker, and do not report the deformation magnitude.

### 6.3. Practical Selection Guidance

The measurements above support the selection guidance of Table 10. All recommendations are restricted to the eleven OpenCV implementations tested at their default parameters under the acquisition conditions of this study.

Illumination was held uniform throughout this study, so it is not covered by these recommendations.

### 6.4. Limitations

The conditions below define the scope within which the reported results hold and indicate where further measurement would extend them:

- Cohort. Seven adults aged 23–28 of a single ethnic background. The cohort is homogeneous and the findings characterise this group; their extension across a wider range of ages, skin tones, and facial morphologies remains to be established.
- Acquisition conditions. Static cameras, uniform controlled illumination, cooperative subjects, and prescribed movements. These are the controlled conditions of an optoelectronic measurement laboratory, and performance in less constrained settings may differ.
- Reference. All tracker results are deviations from the automated Blob Detection reference. Manual annotation confirmed the reference on a sample of 668 drawn marker frames, from which it deviated by 0.52 pixels, which is less than the two annotators deviated from each other (Section 5.1); therefore, deviations of about half a pixel are within the uncertainty of the reference, as they are for manual annotation itself.
- Two-dimensional evaluation. All deviations are per-camera 2D pixel quantities, the 2D tracker being the object of study. Their propagation through triangulation into the deviation of 3D coordinates is left to future work.
- Image scale. The 0.24 mm/pixel factor is a single nominal value for a non-planar surface at varying depth, so millimetre figures are approximate conversions. The pixel-domain quantities on which the comparison rests do not depend on it.
- Statistical resolution. With n=7 independent subjects, the smallest attainable *p*-value of an exact paired test is 0.016, so no pairwise difference between trackers survives correction for multiple comparisons, although the overall ranking is highly consistent across subjects (Kendall’s W=0.89); the trackers are reported as descriptive tiers ordered by mean deviation, not as a ranking.
- Model provenance. The OpenCV Nano and ViT trackers are distributed pretrained models without a peer-reviewed description of their training data or weights; the results characterise these distributed models.
- Viewpoint coverage of the robustness results. The retention experiment used the frontal viewpoint; retention at oblique viewpoints was not measured and may be lower.
- Initialisation. The initial regions were defined by a single operator and the same regions were supplied to every tracker, so the comparison between trackers is unaffected; inter-operator variability was not characterised.

## 7. Conclusions

This study has presented a quantitative evaluation of eleven visual tracking algorithms available in the OpenCV framework and benchmarked their performance for tracking hand-drawn markers on deforming human faces. The comparative analysis utilized a high-density multi-sensor dataset comprising 41,641 frames of full HD video (1920×1080 pixels) captured across seven subjects performing exaggerated phonetic and emotional movements.

The key scientific and technical conclusions for tracking algorithms available in OpenCV 4.10.0 are as follows:

1. Best classical tracker tested: Among the classical filter-based trackers we evaluated, the Channel and Spatial Reliability Tracker (CSRT) was the most dependable choice for long-term coordinate acquisition. CSRT achieved the highest tracking state maintenance success rate of 96.2% in the forty-marker layout, with a mean spatial deviation from the reference of 1.94±1.29 pixels (0.46±0.31 mm) in the four-marker layout and excellent visual stability, though its single-thread throughput is a moderate 56.9 FPS.
2. Lowest per-frame deviation among the neural trackers tested: Among deep learning architectures, the Vision Transformer-based tracker (ViT) recorded the smallest mean deviation of the algorithms evaluated, within a leading tier for which differences are not significant at the subject level, yielding a minimum mean Euclidean deviation from the reference of 1.06±1.03 pixels (0.25±0.25 mm) in the four-marker layout at a high computational throughput of 164.1 FPS. However, its global attention mechanism lacking spatiotemporal constraints resulted in a lower state maintenance success rate of 75.7% in the forty-marker layout due to transient feature switching under extreme local strain.
3. Best retention among the neural trackers tested: The Distractor-Aware Siamese Region Proposal Network (DaSiamRPN) provided the highest temporal stability among neural network-based trackers, maintaining correct coordinate indexing for 92.4% of target nodes in the forty-marker layout, with a mean deviation from the reference of 1.35±1.26 pixels (0.32±0.30 mm) in the four-marker layout. Due to its distractor suppression model, DaSiamRPN showed little feature switching or identity swapping in these sequences, making it the strongest deep learning candidate among those tested for dense tracking pipelines when coordinate identification is critical, though its single-thread throughput of 11.6 FPS limits its real-time CPU applicability.
4. Extreme computational throughput: The Minimum Output Sum of Squared Error (MOSSE) tracker exhibited an exceptional computational throughput of about 12,500 FPS by executing correlation filter operations in the Fourier domain; however, its extreme vulnerability to initialization failures and lack of mechanisms for handling occlusion restrict its utility to highly resource-constrained edge computing environments where speed is prioritized over tracking robustness.
5. Performance limitations and failure modes: Several trackers demonstrated severe limitations in non-rigid sensing environments. In the forty-marker layout, the TLD tracker proved highly unstable, retaining only 17.0% of correctly tracked nodes due to conflicting hypotheses generated by its parallelized modules. GOTURN (30.0%), MOSSE (49.6%), and KCF (64.1%) also exhibited catastrophic tracking failures under transient self-occlusions, despite KCF’s otherwise competitive per-frame deviation. Conversely, the MIL tracker maintained high stability (93.0%, second only to CSRT), but introduced substantial high-frequency spatial jitter and the largest deviation from the reference (5.28±2.57 pixels in the four-marker layout) among stable trackers.
6. Agreement of the reference: The Blob Detection reference and the Point Center Weighted Extractor agreed to 0.28±0.24 pixels (approximately 0.07±0.06 mm) on rigid glued markers and to 0.08±0.05 pixels on hand-drawn markers during deformation. The reference deviated from the centres marked manually by two annotators by 0.52 pixels, less than the annotators deviated from each other (0.64 pixels), which supports the use of the reference as a common baseline for all trackers. As the two extractors share their coarse localisation stage, their agreement characterises the centroid estimate, not an absolute geometric ground truth, and the tracker results are reported as deviations from this reference.
7. Scope of the cohort: These results were obtained from seven adults aged 23–28 of a single ethnic background, recorded under controlled illumination with static cameras; thus, they characterise only these eleven implementations under those conditions. Within the cohort, the relative rankings, deviation distributions, and failure modes were qualitatively consistent across participants and both marker layouts. Recruitment across a broader age range, a wider range of skin tones, and varied illumination is the principal item of future work.

In summary, for applications that require the best trade-off between per-frame deviation and computational speed and in which occlusion is rare, ViT is the strongest of the neural trackers tested in this paper. When correct marker indexing and overall tracking stability are paramount, DaSiamRPN is the preferred deep learning solution among those tested, while CSRT is the most dependable classical tracker.

To address the specific vulnerability of neural architectures to identity swapping in dense layouts, a cooperative hybrid pipeline (ViT+CSRT) was also engineered and evaluated (Section 6.1). By utilizing ViT for high-throughput tracking and CSRT as a drift detection and state correction filter, the hybrid architecture reduced the mis-assignment rate of standalone ViT from 14.42% to 0.28% while retaining 96.2% of the markers, equalling or exceeding CSRT in every sequence, and deviated less than CSRT (1.90 versus 2.37 pixels in the forty-marker sequences and 1.09 versus 1.94 pixels in the four-marker sequences). The pipeline carries the cost of both trackers and its switching criteria have not been validated on held-out data, so cooperative correction is a promising direction for dense non-rigid tracking but not yet a settled improvement.

## Figures and Tables

**Table 1:** Classification of the evaluated OpenCV tracking algorithms by methodology. The year is that of the originating publication, not of the OpenCV integration; ^†^ indicates the year of release of the OpenCV model, for which no peer-reviewed description exists.

Category	Tracker	Core Method	Published
Classical	Boosting	Online AdaBoost classifiers	2006
	MOSSE	Correlation filter (FFT-based)	2010
	MedianFlow	Forward-backward error estimation	2010
	MIL	Multiple instance learning	2011
	TLD	Tracking-Learning-Detection	2012
	KCF	Kernelized correlation filters	2015
	CSRT	Channel and spatial reliability filter	2017
Neural network	GOTURN	Deep regression CNN	2016
	DaSiamRPN	Distractor-aware Siamese network	2018
	Nano	Lightweight Siamese (backbone + neckhead)	2022 ^†^
	ViT	Vision Transformer (ViT)	2023 ^†^

**Table 2:** The two sets of movements performed by participants.

Emotions	Visemes
Wide Smile	PUT
Shocked	FAT
Angry	THINK
Disgust	TIP
	DOLL
	SHE
	ZEAL
	LOT
	RED
	BOOK

**Table 3:** Hardware specifications for the computational latency benchmark.

Operating System	Processor (CPU)	System Memory (RAM)	Architecture
Ubuntu 24.04 (WSL2)	13th Gen Intel(R) Core(TM) i9-13900H 2.60 GHz (Intel Corporation, Santa Clara, CA, USA)	16 GB	x64

**Table 4:** Coordinate deviations and Euclidean distances between the tracking algorithms and the Blob Detection reference; $d_{x}$ and $d_{y}$ are mean absolute component deviations, $d_{x}=\frac{1}{N}\sum_{i}\left|X_{\mathrm{TR},i}-X_{\mathrm{BD},i}\right|$ and likewise for $d_{y}$. All values are conditional on active tracking: frames in which the tracker had lost its marker (Section 4.3) are excluded, so the maxima are bounded by the two-diameter criterion; the last column gives the percentage of frames in which the tracker was active. The ± values are standard deviations pooled over frames; subject-level confidence intervals are given in the text.

Tracker	$d_{x}$ (px)	$d_{x}$ max (px)	$d_{y}$ (px)	$d_{y}$ max (px)	Distance (px)	Distance Max (px)	Distance (mm)	Distance Max (mm)	Active (%)
Boosting	1.39 ± 1.20	21.18	1.62 ± 1.31	21.33	2.35 ± 1.46	27.35	0.57 ± 0.35	6.56	97.5
CSRT	1.24 ± 1.11	16.84	1.25 ± 1.04	19.84	1.94 ± 1.29	25.11	0.46 ± 0.31	6.03	98.5
DaSiamRPN	0.88 ± 1.05	20.13	0.84 ± 0.92	20.84	1.35 ± 1.26	25.04	0.32 ± 0.30	6.01	97.4
GOTURN	2.42 ± 2.18	32.39	1.21 ± 1.90	35.63	2.88 ± 2.72	38.25	0.69 ± 0.65	9.18	68.1
KCF	1.36 ± 1.14	16.12	1.33 ± 1.04	14.09	2.10 ± 1.25	16.87	0.50 ± 0.30	4.05	84.5
MIL	3.47 ± 2.40	24.95	3.31 ± 2.40	19.30	5.28 ± 2.57	26.15	1.27 ± 0.62	6.28	99.3
MOSSE	2.05 ± 1.52	14.98	2.15 ± 1.58	13.09	3.21 ± 1.81	16.25	0.77 ± 0.43	3.90	49.2
MedianFlow	2.56 ± 2.30	24.01	2.20 ± 2.21	20.63	3.75 ± 2.75	27.20	0.90 ± 0.66	6.53	97.7
Nano	1.91 ± 1.85	17.47	1.94 ± 1.68	19.95	3.07 ± 2.05	23.00	0.74 ± 0.49	5.52	98.6
TLD	5.95 ± 4.17	24.31	5.37 ± 3.69	23.20	8.89 ± 4.03	24.32	2.13 ± 0.97	5.84	82.0
ViT	0.64 ± 0.77	22.70	0.71 ± 0.83	21.93	1.06 ± 1.03	26.86	0.25 ± 0.25	6.45	97.2

**Table 5:** Sensitivity to the initial region size on the fourteen frontal four-marker sequences: mean deviation from the reference over active frames (px) and the fraction of frames on which the tracker remained active, for regions of side *s* times the marker diameter.

	s=1.5		s=2.0		s=2.5		s=3.0	
Tracker	Dev.	Active	Dev.	Active	Dev.	Active	Dev.	Active
CSRT	2.31	1.00	2.27	1.00	2.26	1.00	2.12	1.00
ViT	1.14	1.00	1.08	1.00	1.11	1.00	1.09	1.00
DaSiamRPN	1.06	1.00	1.13	1.00	1.19	1.00	1.30	1.00
KCF	2.23	0.57	2.22	0.75	2.38	0.88	2.37	0.90
Nano	1.58	1.00	2.17	1.00	3.05	1.00	3.56	1.00
MedianFlow	3.06	0.98	3.65	0.98	3.91	0.98	4.02	0.99
Boosting	2.78	0.89	2.65	0.97	2.82	0.98	2.56	0.98
MIL	3.77	1.00	3.81	1.00	4.52	1.00	5.85	1.00
MOSSE	3.11	0.05	2.91	0.58	2.97	0.80	3.22	0.71
GOTURN	2.99	0.78	3.01	0.78	3.11	0.79	3.13	0.79
TLD	7.07	0.37	8.53	0.65	9.87	0.69	11.05	0.82

**Table 6:** Per-frame execution latency for one tracking node with OpenCV pinned to a single thread; mean ± standard deviation.

Tracker	Time per Frame (ms)
Boosting	7.15 ± 1.02
CSRT	17.57 ± 1.46
DaSiamRPN	85.90 ± 5.47
GOTURN	38.53 ± 10.54
KCF	2.25 ± 0.35
MIL	24.17 ± 5.21
MOSSE	0.08 ± 0.03
MedianFlow	6.14 ± 1.58
Nano	7.43 ± 1.25
TLD	90.33 ± 11.16
ViT	6.10 ± 0.54

**Table 7:** Performance of the cooperative hybrid pipeline versus standalone baseline trackers, evaluated on the dense forty-marker configuration across thirteen frontal sequences. Final retention and mis-assignment rate are means over the seven participants; the mis-assignment rate denotes the fraction of tracked frames in which a tracked position was closer to an adjacent marker than to its assigned target. The mean deviation is computed over active frames and pooled over frames, as in Table 4; it is larger than in Table 4 because the markers of the dense layout lie close together.

Pipeline	Final Retention (%)	Mis-Assignment Rate (%)	Mean Deviation (px)
ViT alone	75.7	14.42	1.82
CSRT alone	96.2	0.28	2.37
ViT + CSRT hybrid	96.2	0.28	1.90

**Table 8:** The dataset in this paper relative to general single-object tracking benchmarks. Benchmark figures are taken from the cited source publications. For this work, two values separated by a slash refer to the four-marker and forty-marker layouts.

Property	OTB-2015 [76]	VOT2019 [77]	LaSOT [78]	GOT-10k [79]	This Work
Subjects	—	—	—	—	7
Cameras per sequence	1	1	1	1	32 (5 used for deviation, 1 for retention)
Targets per frame	1	1	1	1	up to 4/40
Targets mutually near-identical	no	no	no	no	yes
Target size	large, varied	large, varied	large, varied	large, varied	median 12–13 px (range 5–22 px)
Target contrast	high, varied	high, varied	high, varied	high, varied	high against skin; achromatic, like facial features
Non-rigid target deformation	rare	rare	some	some	all targets; neighbour distance change median 2.3/3.3%, 95th percentile 13.6/21.9%
Occlusion	annotated attribute	annotated attribute	annotated attribute	annotated attribute	marker detected in 97.7/96.6% of frames
Camera motion, illumination change	yes	yes	yes	yes	held constant by design
Annotation	manual boxes	manual boxes	manual boxes	manual boxes	detector reference; 0.28 px from PCWE (rigid), 0.52 px from manual centres (drawn)
Public availability	open	open	open	open	code and data open; videos restricted
Sequences	100	60	1400	10,000	70/13
Frames	≈59,000	≈20,000	≈3.52 million	≈1.5 million	41,641

**Table 9:** The dataset in this paper relative to facial motion capture and facial landmark tracking datasets. Figures for the other datasets are taken from the cited source publications.

Dataset	Subjects	Sequences; Frame Rate	Capture	Content	Annotation	Availability
300-VW [80]	1 per video	114; 30 fps	1 camera, in the wild	uncontrolled	68 landmarks	open
IEMOCAP [81]	10	≈12 h; 120 fps	Vicon, 8 cameras	scripted and improvised dialogue	53 markers, 3D	licence on request
BU-4DFE [82]	101	606; 25 fps	dynamic 3D scanner	posed expressions	3D meshes	research licence
BP4D-Spontaneous [83]	41	328; 25 fps	passive stereo, 2D video	spontaneous expressions	meshes, landmarks, action units	research licence
D3DFACS [84]	10	519; 60 fps	3dMD, 6 cameras	posed action units	3D meshes; FACS-coded	research licence
VOCASET [85]	12	480; 60 fps	3dMD, 18 cameras	speech	registered meshes	research licence
Multiface [86]	13	≈12,200–23,000 frames per subject; 30 fps	40–160 cameras	expressions, speech	tracked meshes	open, non-commercial
This work	7	83; 60 fps	32 cameras (5 used)	expressions, visemes	drawn markers, detector reference	code and data open

**Table 10:** Scenario-based tracker selection for drawn epidermal markers.

Operating Scenario	Recommended	Reason
Frequent self-occlusion	CSRT, DaSiamRPN	Highest retention within the leading deviation tier (forty-marker layout).
Many closely spaced objects	CSRT, DaSiamRPN	High retention and few identity swaps at forty-marker density (0.3% and 1.6% of frames).
Best per-frame deviation	ViT, DaSiamRPN, CSRT, KCF	Leading deviation tier in the four-marker layout; differences within it not resolved with seven subjects; choose on retention and latency instead.
Real time, few targets	KCF, CSRT	Sufficient throughput at competitive deviation.
Embedded/low-power	MOSSE, ViT, Nano	Lowest single-thread latency overall (MOSSE, 0.08 ms) and among the neural trackers (ViT 6.1 ms, Nano 7.4 ms).
Not recommended	TLD, GOTURN	Retention below 35% under transient self-occlusion (forty-marker layout).

## Data Availability

The source code of the evaluation, including the benchmark application, the benchmark runner, and the analysis scripts, is openly available at https://github.com/PhotonicTree/TrackerApp (accessed on 15 October 2026); the application can be used to evaluate the trackers on other videos. The coordinate data derived from the recordings (the initial marker regions, reference detections, outputs of all trackers in every frame, and manual marker centres, with subjects identified by code) are released with the code under the Creative Commons Attribution 4.0 licence; the deviation, failure, and retention results reported here can be recomputed from them with the released scripts. The video recordings are not publicly available because the participants did not consent to their public release.

## Appendix A

The Blob Detection algorithm implemented within the open-source OpenCV library [17] was used to extract the baseline coordinate trajectories that serve as the reference throughout this study. Its parameter values are listed in Table A1; the area limits are set for each video from the diameters of its markers in the first frame so that they follow the apparent marker size in each camera view. All operational parameters not explicitly listed here were maintained at the native OpenCV default values.

**Table A1:** OpenCV Blob Detection parameter values used in this study; $d_{10}$ and $d_{90}$ are the 10th and 90th percentiles of the marker diameters of the video, in pixels.

Parameter	Value
thresholdStep	1
minThreshold	20
maxThreshold	150
minRepeatability	4
minDistBetweenBlobs	10
filterByColor	true
blobColor	0
filterByArea	true
minArea	$\max\left(8,\;0.4\;\pi d_{10}^{2}/4\right)$
maxArea	$2.5\;\pi d_{90}^{2}/4$
filterByCircularity	true
minCircularity	0.50
maxCircularity	1.00
filterByConvexity	true
minConvexity	0.75
filterByInertia	true
minInertiaRatio	0.10
maxInertiaRatio	1.01
